# Complement C1q is a key player in tumor-associated macrophage-mediated CD8^+^ T cell and NK cell dysfunction in malignant pleural effusion

**DOI:** 10.7150/ijbs.100607

**Published:** 2024-11-04

**Authors:** Feng-Shuang Yi, Xin Qiao, Shu-Feng Dong, Qing-Yu Chen, Rui-Qi Wei, Ming-Ming Shao, Huan-Zhong Shi

**Affiliations:** 1 Department of Respiratory and Critical Care Medicine, Beijing Institute of Respiratory Medicine and Beijing Chao-Yang Hospital, Capital Medical University, Beijing 100020, China.; 2 Medical Research Center, Beijing Institute of Respiratory Medicine and Beijing Chao-Yang Hospital, Capital Medical University, Beijing 100020, China.; 3 Department of Respiratory and Critical Care Medicine, Tianjin Chest Hospital, Tianjin University, Tianjin 300222, China.

**Keywords:** malignant pleural effusion, C1q, macrophages, metabolomics, NK cells

## Abstract

Macrophages play a crucial role in malignant pleural effusion (MPE), a frequent complication of advanced cancer. While C1q^+^ macrophages have been identified as a pro-tumoral cluster, direct evidence supporting the role of C1q-mediated macrophages remains to be elucidated. This study employed global and macrophage-specific knockout mice to investigate the role of C1q in MPE. The data demonstrated that C1q deficiency in macrophages suppressed MPE and prolonged mouse survival. scRNA-seq analysis of the C1qa^-/-^ mouse MPE model revealed that C1q deficiency significantly decreased the proportion of M2 macrophages in MPE. *In vitro* experiments suggested that C1q expression was gradually upregulated during M2 polarization, which was C1q-dependent, as was antigen presentation. Deficiency of C1q in macrophages rescued the exhausted status of CD8^+^ T cells and enhanced the immune activity of CD8^+^ T cells and NK cells in both MPE and pleural tumors. Cell-to-cell interaction analysis demonstrated that C1q deficiency attenuated the immunoinhibitory effects of macrophages on NK cells by downregulating the CCR2-CCL2 signaling axis. Metabolomic analysis revealed significantly elevated hippuric acid levels in C1q-deficient mouse MPE. Treatment with either hippuric acid or a CCR2 antagonist inhibited MPE and tumor growth, with an even more pronounced effect observed when both treatments were combined.

## Introduction

Malignant pleural effusion (MPE) is a frequent complication of advanced cancer 1, accounting for 15% to 35% of exudative pleural effusions 2. The overall survival (OS) of MPE patients ranges from 3 to 12 months 3, 4. The primary mechanism of MPE formation is the increase in vascular permeability, usually associated with the lymphatic drainage obstruction at the pleura and/or mediastinum 2. Furthermore, the inflammatory signaling network and immune cell regulation in the pleural cavity are key participants in the formation of MPE 1, 5, 6. In addition to tumor cells, MPE contains large numbers of immune cells including lymphocytes, macrophages, and granulocytes 7-10, all of which function in the occurrence and development of MPE; however, the exact mechanism remains incompletely elucidated.

Tumor-associated macrophages play vital roles in the tumor environment. It is currently believed that a pro-tumoral differentiated monocyte population exhibiting an M2-like signatures is thought nowadays to be one of the key factors contributing to tumor promotion 11. Our research has revealed a significantly higher presence of CD206^+^ macrophages (M2 macrophages) in MPE compared to non-MPE, suggesting that CD206^+^ macrophages are a promising candidate for the lung cancer-originated MPE diagnosis 12. Furthermore, macrophages with M2 signatures are associated with even worse prognosis in lung adenocarcinoma (LUAD) patients 13. In recent years, an increasing number of signature genes, such as TREM2, SSP1, APOE, and C1Q, have been identified to classify macrophages into novel subsets beyond the traditional M1 or M2 categories 14-16.

Previous research has established the presence of complement proteins can be detected in MPE 17. C1q, the initial recognition molecule of the classical complement pathway 18, is an 18-subunit protein consisting of C1QA, C1QB, and C1QC in equal molar ratios 19. Beyond its role in the immune tolerance maintenance and the apoptotic cell clearance 20, C1q directly influences cell differentiation, adhesion, migration, and proliferation in various cell types, participating in numerous immune-related physiological and pathological processes 21-23. In the context of cancer, studies have associated C1q with the progression of diverse malignancies, including lung cancer, mesothelioma, breast cancer, renal clear cell carcinoma, melanoma, and colon cancer 16, 22-24.

Recent advancements in single-cell mRNA sequencing (scRNA-seq) have unveiled C1q acted as a distinctive marker for identifying a novel macrophage subset. scRNA-seq analyses have demonstrated that the C1q^+^ macrophages and other immune cells interactions contribute to immunosuppression, poor prognosis in cancer patients, and suboptimal responses to immunotherapies 16, 25. However, the precise role of C1q as a pro-tumor or anti-tumor factor remains controversial and may vary depending on the specific tumor microenvironment.

Recent investigations have demonstrated that the C1q levels in pleural effusion can differentiate tuberculous pleural effusion (TPE) from non-TPE^26^, ^27^, exhibiting higher diagnostic accuracy than tumor necrosis factor (TNF)-α and IL-6. However, relevant reports regarding the role of C1q in MPE needs further investigations. In contrast to most other complement molecules, which are primarily synthesized by the liver, C1q is mainly secreted by cells derived from myeloid precursor cells, including macrophages 19 and immature dendritic cells (DCs) 19, 28. Recently, C1q^+^ macrophages were found to lead to immunosuppression in a fatty acid metabolic reprogramming-dependent manner in MPE 29. The researchers utilized MHC-II/HLA-DR^+^CX3CR1^+^ and MHC-II/HLA-DR^-^CX3CR1^-^ to define and isolate C1q^+^ and C1q^-^ macrophages, respectively. Subsequently, these "C1q^+^" and "C1q^-^" macrophages were employed in their further investigation. However, the direct evidence to verify the internal role of C1q and the corresponding mechanism in MPE using C1q-deficient mice remains to be elucidated.

This study discovered that C1q functioned in MPE independently of the classical complement pathway, participating in antigen presentation and macrophage polarization. Data from scRNA-seq and flow cytometry of cells from C1q-deficient mice revealed that C1q^+^ macrophages promote MPE by establishing an immunologically inhibitory tumor environment. This process was mediated by the regulation of natural killer (NK) cells through CCR2 signaling and the presence of hippuric acid in pleural effusion.

## Materials and Methods

### Study populations and sample processing

The research received ethical approval from the committees of Beijing Chao-Yang Hospital, Capital Medical University (2021-ke-9). Informed consent was obtained in writing from participants involved in the study. Pleural effusion patients were recruited at Beijing Chao-Yang Hospital. Patients who had undergone any invasive procedures involving the pleura or received chest trauma within three months prior to hospitalization; and who had undergone any anti-tuberculosis, antitumor, glucocorticoids, or other non-steroidal anti-inflammatory treatment were excluded. The diagnosis of MPE was made if malignant cells were detected in the pleural fluid (PF) and/or through pleural biopsy specimens. Diagnostic thoracentesis was performed to collect PF from each participant. Concurrently, blood samples from the periphery were also gathered, together with PF, they were promptly brought to researchers under refrigeration at 4° C and subjected to centrifugation at 400 g for 10 minutes under the same temperature. Nucleated cells were separated using a Ficoll-Paque gradient technique (Pharmacia, Uppsala, Sweden), the cell pellets were assessed within one hour, while the supernatant was preserved at -80 °C for future applications.

### Cell lines

MC38, the cell line derived from mouse colon adenocarcinoma, was gifted by Dr. G.T. Stathopoulos who worked for University of Patras, Rio Patras, Greece. LLC, the murine Lewis lung carcinoma cell line, BEAS-2B, the human bronchial epithelial derived cell line, and A549, the human non-small cell lung carcinoma (NSCLC) derived cell line, all of which were sourced from the American Type Culture Collection, Manassos, VA. Cells were maintained in Dulbecco's modified Eagle's medium (DMEM) that was enriched with 10% heat-inactivated fetal bovine serum (FBS), 100 U/ml Penicillin, 100 mg/ml Streptomycin, 4 mM L-glutamine, and 4.5 g/L HEPES in 5% carbon dioxide at 37 ° C.

### Mice

The mouse related protocols in the current study have adhered to the guidelines approved by the Institutional Animal Care and Use Committee of the Capital Medical University (AEEI-2021-290, AEEI-2023-237). Wild-type (WT) C57BL/6 mice were purchased from Beijing Vital River Laboratory Animal Technology (Beijing, China). C1qa^fl/fl^Lyz2-Cre (conditional knockout (CKO) of C1qa in macrophages) mice were bred by crossing C1qa^fl/fl^ and Lyz2-Cre transgenic mice. C1qa^fl/fl^, Lyz2-Cre, OT-I mice (CD8^+^ T cell transgenic mice expressing a TCR recognizing the K^b^ associated epitope SIINFEKL which is derived from OVA) and OT-II mice (CD4^+^ T cell transgenic mice expressing a TCR recognizing the I-A^b^ associated epitope ISQAVHAAHAEINEAGR which is derived from OVA) were purchased from Cyagen Biotech Co., Ltd (Suzhou, China). The C1qa globally knockout (KO) mice (C1qa^-/-^) were kind gifts from Chao Xiong (Fuwai Hospital, China). Mice were kept in an air-conditioned room with the temperature 23-25° C and the humidity 40-70% under a 12-h dark-light cycle. All mice were used at 6-8 weeks of age.

### Murine models and sample processing

Murine MPE models were established by intrapleural injection of 1.5×10^5^ LLC, or MC38 cells in each mouse as previously described 10, 30-32. Mice were euthanized by CO_2_ asphyxiation fourteen days post LLC cell injection or, ten days post MC38 cell injection and MPE, pleural tumors and blood samples were obtained for subsequent experiments. The pleural tumors were cut into small pieces with ophthalmic scissors to incubate with digestion solution (130 ug/mL DNase I, 130 U/mL Collagenase Type IV, in Medium/RPMI 1640) for 1 hour at 37℃. The digested samples were screened with an 80-100 μm filter, the passed-through samples and MPE cell pellet were subjected to Ficoll-Paque gradient centrifugation to obtain nucleated immune cells.

To verify the effects of hippuric acid and RS504393 on MPE, hippuric acid and RS504393 were freshly dissolved in dimethyl sulfoxide (DMSO), diluted into a solution with DMSO (10%), PEG400 (40%), Tween-80 (5%), and saline (45%) in sequence. Then mice were treated with hippuric acid (0.14 mg/kg/day) 33 and/or RS504393 (6 mg/kg/3 days) 34 (purchased from MedchemExpress) by intraperitoneal injection after the MPE model was established.

### Flow cytometry

The antibodies (Abs) for flow cytometry analysis including anti-human C1q mAb was purchased from Quidel Corporation; anti-human CD3, CD15, CD19, CD45, CD14 mAbs, anti-mouse CD45, CD3, CD8, LAG-3, TIM-3 and PD-1 mAbs were purchased from eBioscience; anti-human HLA-DR, CD86, CD206, anti-mouse CD45, CD3, CD8, NK1.1, F4/80, CD11b, Ly6C, CX3CR1, MHC II, CD86, CD206, and Goat anti-Rat IgG (H+L) Cross-Adsorbed Secondary Antibody, Alexa Fluor™ 488 were purchased from Invitrogen. For staining of human C1q, cells were incubated with the C1q mAb at 4°C for 30 minutes, then the Alexa Fluor 488 goat anti-rat IgG were added and incubated at 4°C for 30 min. For staining of cell surface receptors including human CD3, CD15, CD19, CD45, CD14, HLA-DR, CD86, CD206, mouse CD45, CD3, CD8, F4/80, CD11b, Ly6C, CX3CR1, MHC II, CD86, LAG-3, TIM-3 and PD-1. The corresponding fluorescent Abs were incubated with the cells at 4°C for 15 minutes. For intracellular staining of transcription factors or cytokines, cells were suspended and fixed with fixation/permeabilization solution (BD Biosciences) for 25 minutes, and then anti-CD206, IFN-γ, IL-17, FOXP3, TNF-α, GZMB, and Perforin mAbs were added and incubated for 30 minutes, and then washed by permeabilization buffer (BD Biosciences). Finally, cells were resuspended in PBS and subjected to flow cytometry (BD Biosciences) for further analysis.

### Survival analysis

Mice exhibiting MPE were closely observed, humanely euthanized, and their cases were documented as part of the Kaplan-Meier survival analysis at the point they showed severe illness and were nearing death. The comparison of overall survival was performed by a pairwise log-rank test (GraphPad Software).

### Antigen presentation by macrophage *in vitro*

Immune complexes (ICs) consisting of IgG and OVA were formed by mixing OVA that was linked to Alexa Fluor 647 (Invitrogen) with polyclonal rabbit anti-OVA IgG (Sigma) for 30 minutes at a temperature of 37 °C. After that, macrophages isolated from the MPE of C1qa^fl/fl^ and C1qa^fl/fl^Lyz2-Cre mice were exposed to the OVA ICs for an hour. Following a washing step, these macrophages were then incubated with OT-I or OT-II T cells (pre-labeled with CFSE). The levels of proliferation in CD8^+^ T cells and CD4^+^ T cells were assessed three days later using flow cytometry.

### Immunoturbidimetric assay

Immunoturbidimetric method was used to determine levels of C3 and C4 by commercial kits (3V biotech, Weifang, China), and values were expressed as mg/dL.

### Enzyme-Linked Immunosorbent Assay (ELISA)

Hippuric acid ELISA Kit (SPS-21964, SAIPEISEN BIOLOGY, Shanghai) was used to determine hippuric acid level in MPE according to the manufacturer's instructions.

### Luminex assay

The levels of IFN-γ, TNF-α, CCL2, CCL3, CCL4, CCL7 and CCL8 in mouse MPE were determined using Luminex assay with Mouse Cytokine & Chemokine Panel 1A (36 plex) (Invitrogen), the concentration of GZMB was determined with Mouse ProcartaPlex Simplex Kit (EPX010-26074-901). Luminex 200 was used to detect and calculate the concentrations.

### Preparation of conditional medium

When the LLC, A549, and BEAS-2B cells were cultured to the logarithmic growth phase, 5×10^6^ cells were inoculated into a 75cm^2^ culture flask. After twenty-four hours of culture, the medium was changed into serum-free medium. Forty-eight hours later, the cell supernatant was centrifuged and filtered by a 0.22 μm sterile filter, and then stored at -80° C for later cell culture.

### RNA extraction, reverse transcription, and quantitative RT-PCR

TRIzol (Thermo Fisher Scientific) were used to extract the total RNA. The RNA with the amount of 1000 ng/20 μL/test underwent processing for cDNA synthesis utilizing the Reverse Transcription Kit (Takara) utilizing random primers. To quantify the mRNA expression levels, one microliter of the synthesized cDNA was analyzed using SYBR Green I Master (Roche, Basel. Switzerland), and the calculations of the results were performed applying the 2^-ΔΔCT^ methodology, with GAPDH serving as the control. Each sample underwent testing in triplicate.

### Murine bone marrow-derived macrophage (BMDM) isolation and polarization

Bone marrow (BM) was flushed from femurs and tibia of WT, C1qa^fl/fl^Lyz2-Cre or C1qa^fl/fl^ mice on C57BL/6 background. BM cells were cultured in DMEM containing 10% heat-inactivated FBS, 100 U/ml Penicillin, 100 mg/ml Streptomycin, 4 mM L-glutamine, 4.5 g/L HEPES and 20 ng/mL macrophage colony-stimulating factor (M-CSF) in 5% CO_2_ at 37°C. The medium was replenished every three days to support the maturation of M0 macrophages. On the seventh day, the M1 phenotype was induced by LPS (100 ng/mL, Sigma), and IFN-γ (40 ng/mL, Peprotech), while recombinant murine IL-4 (10 ng/mL, Peprotech) was utilized to promote the M2 phenotype. To investigate the expression of markers related to antigen presentation and the polarization of macrophages, cells were treated with hippuric acid (MedchemExpress) for periods of 3 and 10 days, respectively.

### Immunofluorescence

Tumors located in the pleura were preserved using 4% paraformaldehyde and subsequently incorporated into paraffin. Thin sections measuring 5 μm were then produced from the paraffin blocks. These sections underwent incubation with monoclonal antibodies for immunofluorescence, specifically targeted against CD31 (Servicebio, Wuhan). Imaging was performed utilizing a Leica TCS SP5 microscope.

### Cell transfection assay

The C1qa, C1qb, and C1qc-specific siRNAs (siC1qa, siC1qb, siC1qc, we used the mixture of three with equal molar ratio), negative control and 5-Fam (all from RiboBio) were introduced into murine BMDM using Lipofectamine RNAiMAX (Life Technologies, Carlsbad, CA).

### Apoptosis assay

Murine BMDMs before and after transfection were suspended in DMEM containing 10% heat-inactivated FBS and cultured at 2×10^6^ cells/mL. Twenty-four hours later, cells were collected and the apoptotic fractions were detected and analyzed using an FITC Annexin V Apoptosis Detection Kit (BD Biosciences) adhering to the manufacturer's instruction.

### Phagocytosis assay

Mouse macrophages isolated from C1qa^fl/fl^Lyz2-Cre or C1qa^fl/fl^ mice were suspended in DMEM containing 10% heat-inactivated FBS, cultured at 2×10^6^ cells/ml, and collected 24, 48 and 72 hours later. Phagocytosis capacity was detected using a pHrodo™ Green E. coli BioParticles™ Conjugate (Invitrogen) adhering to the manufacturer's instruction.

### Single-cell RNA sequencing analysis

The cells with nuclei extracted from murine MPE and pleural tumors were derived following the methodologies specified for mouse models and sample analysis, subsequently subjected to trypan blue staining (Sigma, Shanghai, China) and assessed under a microscope for cell viability. Cells exhibiting a viability rate of 80% or more were selected for further processing. The resultant single-cell suspensions were adjusted to 1 × 10^5^ cells/mL in concentration in PBS and introduced into microfluidic platforms, while single-cell RNA sequencing libraries were generated in accordance with the Singleron GEXSCOPE® protocol utilizing the GEXSCOPE® Single-Cell RNA Library Kit as well as the Singleron Matrix® Automated single-cell processing system (Singleron Biotechnologies). The resulting libraries were adjusted to 4 ng/µL in concentration and then combined for the sequencing process. The pooled libraries underwent sequencing on the Illumina novaseq6000 platform with 150 bp paired-end reads.

The resulting reads were aligned to the reference genome GRCh38.93. Outcomes from the Cell Ranger analysis provided UMI counts associated with each gene across all cells for each sample employing all mapped reads. Data analysis objects were constructed using the R package Seurat (v4.0.0). Initially, genes expressed in fewer than 200 cells (about 0.1% of the total) were filtered out. Subsequently, cells were excluded if they demonstrated expression of fewer than 200 genes, more than 6000 genes, or over 10% of mitochondrial genes. The DoubletFinder (v2.0.1) was then applied to identify and remove doublets from subsequent analysis. Finally, the graph-based clusters were annotated with known biological cell types utilizing canonical marker genes (v.3.12). by referring to the results of SingleR (v.3.12) as well. The FindAllMarkers and FindMarkers functions were used to determine the differentially expressed genes between a certain cell subgroup and all other clusters or a specific cluster with the following signatures (i) genes expressed in at least 30% of the cells and (ii) log2(fold change) > 1. Interactions were calculated based on ligand and receptor expression on certain two types of cells. CellChatDB (http://www.cellchat.org/) served as references for ligand-receptor interacting pairs. An expression matrix derived from the data slot of the Seurat object of each cluster was used as input for CellChat (0.5.5). The probability values were used to assess and determine potential cell-cell interactions. Interactions between receptors and ligands for which P < 0.05 were considered as statistically significant.

### Bulk RNA sequencing analysis

Total RNA was extracted from immune cells utilizing TRIzol (Invitrogen, Waltham, MA, USA) adhering to the instructions provided by the manufacturer. Subsequently, the concentration and integrity of RNA were meticulously determined using the RNA Nano 6000 detection kit. The NEBNext®UltraTM RNA Library Prep Kit (NEB, Ipswich, MA, USA) was used to prepare the sequencing libraries. The libraries were then proceed and sequenced on the Illumina NovaSeq 6000 platform (Illumina, San Diego, CA, USA), producing high-quality paired-end reads with a double-ended read length of 150 bp. The resulting reads were accurately aligned with the GRCh38 reference genome. To identify differential expressed genes between the two groups, we employed the DEGseq2 package (v.3.18). Significant differential expression was determined with an error discovery rate (FDR) under 0.05 and a log2 fold change of ≥ 1.

### Metabolomics analysis

The MPE supernatant samples previously stored at -80 °C were removed and gently thawed in an ice-water mixture. Metabolomics assays were accomplished by Shanghai Luming Biological Technology Co., Ltd. (Shanghai, China). Following mixing of the sample (100 μL) with the protein precipitator methanol-acetonitrile (V:V = 2:1, inclusive of mixed internal standard, 400 μL, 4 μg/mL), the sample underwent extraction, centrifugation, and filtering. Subsequently, the supernatant was transferred to LC injection vials. Mixing all sample extracts in equal volumes were used to prepare the quality control samples.

The metabolic profiling was conducted by ACQUITY UPLC I-Class plus (Waters Corporation, Milford, USA) fitted with Q-Exactive mass spectrometer which was equipped with heated electrospray ionization (ESI) source (Thermo Fisher Scientific, Waltham, MA, USA) in both positive and negative ion modes. The mass range was from 70 to 1050 m/z.

The raw LC-MS data were processed using Progenesis QI V2.3 (Nonlinear, Dynamics, Newcastle, UK) software to filter baseline, identify and integrate peak, correct retention time, align and normalize peak. The Human Metabolome Database (HMDB), Lipidmaps (v2.3), Metlin, and self-built databases were used to identify compounds based on precise mass-to-charge ratio (M/z), secondary fragments, and isotopic distributions. The extracted data underwent further processing to eliminate any peaks with missing value rate over 50% (ionic strength = 0) within the group. Half of the minimum value were used to replace the zero values, and compounds were filtered based on their qualitative results. Additionally, compounds with a database match score below 36 out of a total of 80 were deemed inaccurate and excluded. Finally, the positive and negative ion data were consolidated into a comprehensive data matrix.

### Statistics

Data were shown as mean ± standard error. Statistical differences between groups were determined using Student's *t*-test, or one-way ANOVA followed by Bonferroni test for grouped comparisons. Kaplan-Meier method was used to analyze the survival, and log-rank test was used to determin the statistical differences. Graph-Pad Prism or SPSS statistical software was used for statistical calculations, and there was statistical significance when the P value is under 0.05.

## Results

### C1q deficiency inhibited mouse MPE formation

To dig out the potential role of complement C1q in the pathogenesis of MPE, we initially compared the expression levels of C1qa, C1qb, and C1qc within nucleated cells between mouse MPE and blood using quantitative polymerase chain reaction (qPCR). The results demonstrated that all three C1q subunits exhibited higher expressions in MPE nucleated cells than those in blood (Supplementary Fig. 1A). However, upon measuring the levels of C3 and C4, the downstream effector elements in the classical complement pathway, we observed that C3 and C4 were significantly lower in MPE than in blood (Supplementary Fig. 1B). These findings led us to hypothesize that complement C1q may be involved in MPE formation through a mechanism which is classical complement pathway-independent.

**Figure 1:**
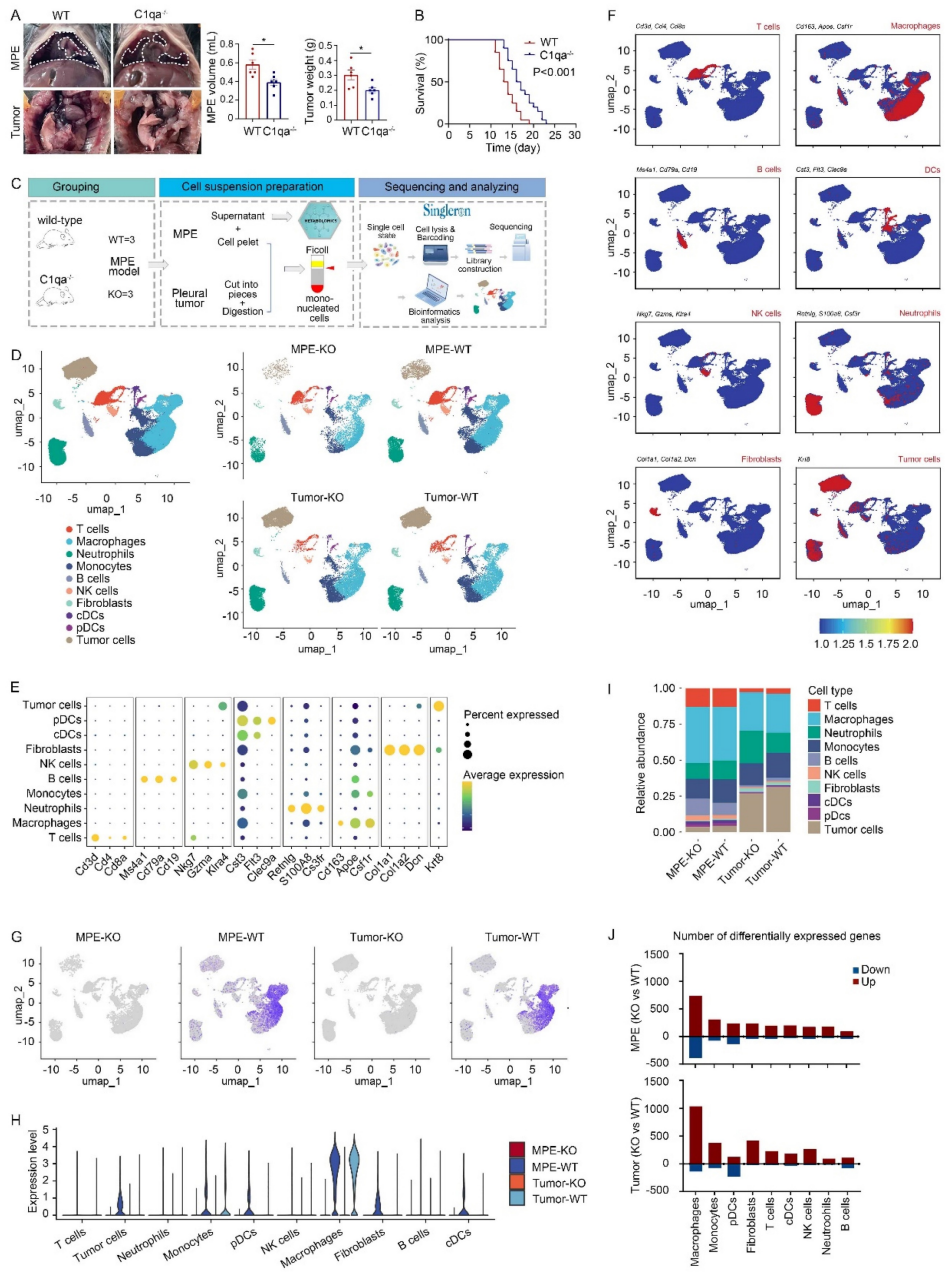
** C1qa deficiency in mice suppressed MPE development.** (A) Anatomical images of pleural effusion and tumors of WT and C1qa^-/-^ mice. receiving intrapleural injection of LLC cells (left panel). Comparisons of MPE volume (each n = 6) and pleural tumor mass (each n = 6) between WT and C1qa^-/-^ mice (middle two panels). (B) Life span analysis was carried out in WT and C1qa^-/-^ mice receiving intrapleural injection of LLC cells (each n = 20) (right panel). (C) Mononucleated cell scRNA-seq profiling and MPE supernatant metabolomic workflow. (D) UMAP plot of the scRNA-seq data of the pleural effusion and tumor tissue from WT mice or C1qa^-/-^ mice. (E) A dot plot showed the marker gene expression in the corresponding cell cluster. (F) The expression of the marker genes was shown by a color gradient in the UMAP plot. (G) The expression of C1qa was shown by a color gradient in the UMAP plot. (H) A violin plot showed the expression of C1qa in the corresponding cell cluster. (I) Bar graphs showed each cell cluster proportion. (J) The differentially expressed gene number between C1qa^-/-^ mice and WT mice in MPE (upper panel) or tumor tissues (bottom panel). Data were presented as means ± SEM. *P < 0.05; the comparisons were determined by Student's *t*- test or pairwise log-rank test (survival analysis). KO, C1qa^-/-^ mice; WT, WT mice.

To examine the role of C1q in mouse MPE, we initially obtained C1qa globally knockout (C1qa^-/-^, also denoted as KO) mice. LLC cells were injected intrapleurally into C1qa^-/-^ and WT mice. Approximately 14 days post-cell injection, MPE and pleural tumors were collected and analyzed. The results demonstrated a reduced volume of MPE volume and smaller mass of pleural tumors in C1qa^-/-^ mice compared to WT ones (Fig. 1A). Furthermore, the C1qa^-/-^ mice displayed a longer life span than the WT ones (Fig. 1B). Comparable phenomenon was observed in mice receiving MC38 cells (Supplementary Fig. 1C). Bulk RNA sequencing data of immune cells in MPE revealed that C1qa depletion led to the enrichment of pathways related to immune response activation, cytokine production, lymphocyte differentiation, and T cell activation (Supplementary Fig. 1D).

To provide a comprehensive interpretation of C1q-mediated immune responses during MPE formation, we collected MPE and pleural tumors from C1qa^-/-^ and WT mice, isolated mononucleated cells, and proceeded with scRNA-seq using the Singleron platform (Fig. 1C). Uniform manifold approximation and projection (UMAP) was used to perform unsupervised clustering, and the obtained 72,873 cells were divided into 22 clusters, the vast majority of which were immune cells (Ptprc positive) (Supplementary Fig. 2A and B). Based on the classical marker gene expression, ten cell types were identified: T and B lymphocytes, NK cells, neutrophils, monocytes, macrophages, classical dendritic cells (cDCs), plasmacytoid dendritic cells (pDCs), fibroblasts, and tumor cells (Fig. 1D-F, Supplementary Fig. 2C-E). Significantly reduced C1qa expression, but not C1qb and C1qc expression, was observed in both MPE and tumor tissue of KO mice (Fig. 1G and Supplementary Fig. 2F), indicating effective C1qa disruption in C1qa^-/-^ mice. In both MPE and tumor tissues, C1qa was primarily expressed in macrophages (Fig. 1H). No significant alterations were detected in the cell type proportion in C1qa^-/-^ mice compared to WT mice, and flow cytometry confirmed these results (Fig. 1I and Supplementary Fig. 2G). However, the analysis of differentially expressed gene number between C1qa^-/-^ and WT mice showed that C1qa KO caused significant transcriptional changes in macrophages, suggesting that C1q primarily influences MPE formation by affecting key macrophage functions (Fig. 1J). Next, the peripheral blood and MPE cells from MPE patients were analyzed by flow cytometry. Consistent with the mouse model results, C1q levels in MPE were the highest in macrophages (Supplementary Fig. 3A). Compared with peripheral blood, C1q expression level in nucleated cells was higher in MPE than that in blood. Notably, the expression level of C1q in macrophages and DCs was significantly higher in MPE than that in blood. However, there was no significant differences between MPE and blood as to granulocytes, T cells, and B cells (Supplementary Fig. 3B). Expression of C1qa was not associated with survival in all LUAD patients of The Cancer Genome Atlas (TCGA) cohort or in patients with lymph node or distant metastases. Interestingly, after normalization based on macrophage fractions, patients with high C1qa expression exhibited significantly poorer overall survival (OS) (Supplementary Fig. 4). Therefore, we paid our attention to the role of C1q in macrophages.

### C1q deficiency in macrophages inhibited MPE and macrophage antigen presentation

**Figure 2:**
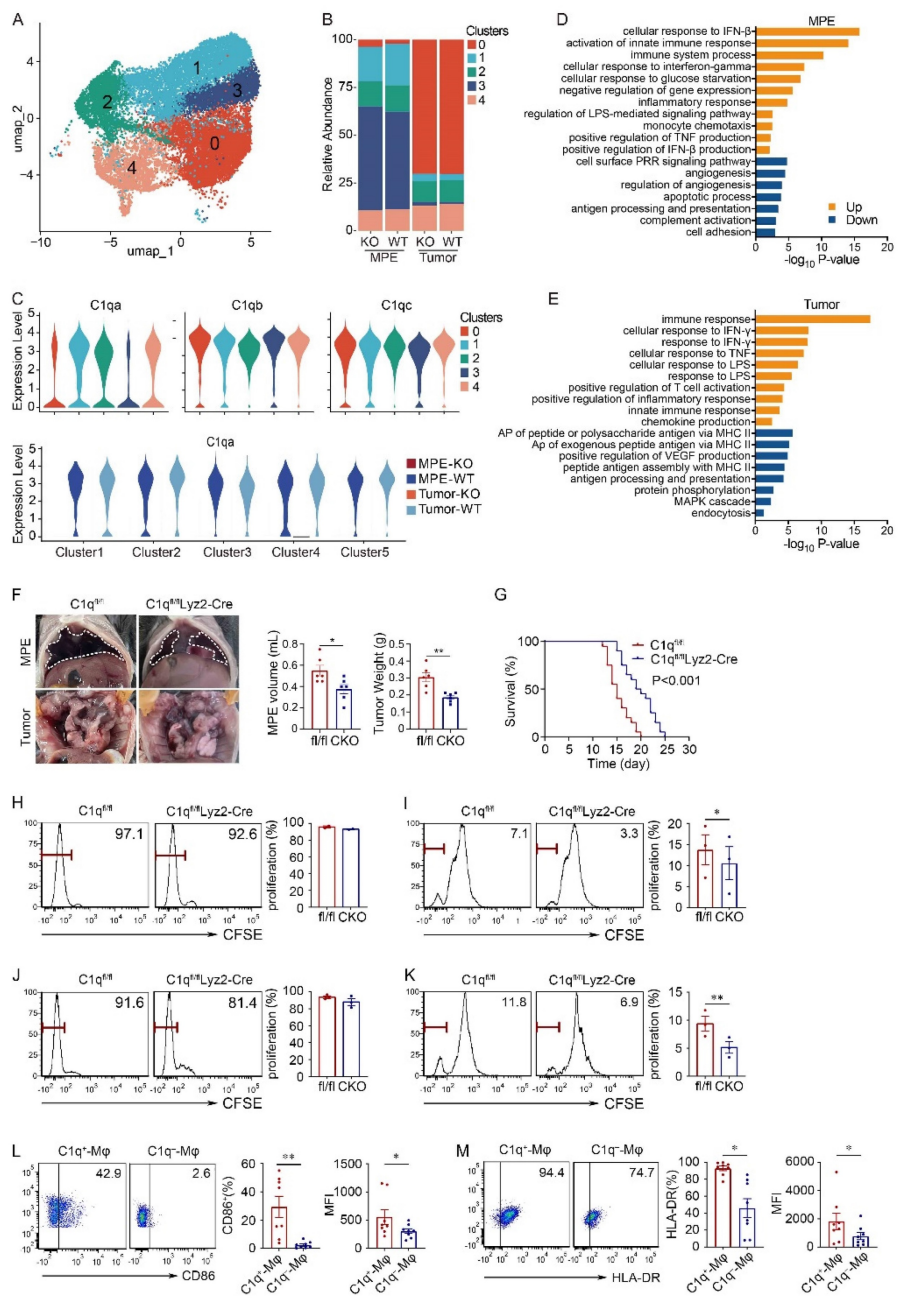
** C1q deficiency in macrophages inhibited MPE and macrophage antigen presentation.** (A) UMAP plot of the scRNA-seq data of macrophages. (B) Bar graphs showed the proportion of each macrophage cluster. (C) A violin plot showed the expression of C1qa in the corresponding cell cluster. (D) Bar graph showed the enrichment pathways in the up- or down-regulation genes of MPE between C1qa^-/-^ and WT mice. (E) A bar graph showed the enrichment pathways in the upregulated or downregulated genes of tumor between C1qa^-/-^ and WT mice. AP, antigen-presenting. (F) Anatomical images of pleural effusion and tumors of C1q^fl/fl^ and C1q^fl/fl^Lyz2-Cre mice receiving an intrapleural injection of LLC cells a (left panel). Comparisons of MPE volume (each n = 6) and pleural tumor mass (each n = 6) between C1q^fl/fl^ and C1q^fl/fl^Lyz2-Cre mice (middle two panels). (G) Life span analysis was performed in C1q^fl/fl^ and C1q^fl/fl^Lyz2-Cre mice receiving intrapleural injection of LLC cells. The OS of the two groups was analyzed using the Kaplan-Meier method and a pairwise log-rank test (each n = 20) (right panel). (H, I) Macrophages from C1q^fl/fl^ and C1q^fl/fl^Lyz2-Cre MPE mice receiving an intrapleural injection of LLC cells (each n = 3) that were incubated with OVA immune complex (IC) for one hour were washed and incubated with CFSE-labeled spleen lymphocytes. CD4^+^ T cell (H) and CD8^+^ T cell (I) proliferation was tested after three days by flow cytometry. (J, K) Macrophages from C1q^fl/fl^ and C1q^fl/fl^Lyz2-Cre MPE mice receiving intrapleural injection of MC38 cells (each n = 3) that were incubated with OVA IC for one hour, then were washed and incubated with CFSE labeled spleen lymphocytes. CD4^+^ T cell (J) and CD8^+^ T cell (K) proliferation was measured after three days by flow cytometry. (L) The representative flow cytometric dot plots (left panels) and statistical comparisons (right panels) of CD86 level in C1q^+^-Mφs compared with C1q^-^-Mφs in patients' MPE (n = 20). (M) The representative flow cytometric dot plots (left panels) and statistical comparisons (right panels) of HLA-DR level in C1q^+^-Mφs compared with C1q^-^-Mφs in patients' MPE (n = 20). Data were presented as means ± SEM. *P < 0.05, **P < 0.01, ***P < 0.001; the statistical comparisons were determined by Student's *t*-test or pairwise log-rank test (survival analysis).

Macrophages were divided into five subclusters, all of which exhibited similar proportions in the KO and WT groups. All subclusters highly expressed comparable levels of C1qa, C1qb, and C1qc, and demonstrated successful C1qa disruption in the KO group (Fig. 2A-C). Consequently, subsequent analyses and experiments were conducted on macrophages as a whole. Compared with WT mice, macrophages in the C1qa^-/-^ mice exhibited enrichment in immune response, interferon (IFN), and TNF production signaling pathways. Conversely angiogenesis and antigen-presenting association pathways were downregulated in both MPE and tumor tissues (Fig. 2D and E).

To dig out the function of C1q^+^ macrophages in the development of MPE, this study introduced a conditional macrophage-specific genetic ablation of C1qa in C1qa^fl/fl^Lyz2-Cre mice (also referred to CKO in the text), as C1q is predominantly expressed in macrophages (Supplementary Fig. 5A). Compared with C1qa^fl/fl^ mice (also denoted as fl/fl in the text), C1qa CKO mice exhibited reduced volume of MPE, decreased weight of pleural tumors, and extended time of survival, consistent with findings from C1qa globally KO mouse MPE models (Fig. 2F and G). Analogous results were observed in mice receiving MC38 cells (Supplementary Fig. 5B and C). Furthermore, a comparison of survival between C1qa CKO and C1qa^-/-^ mice indicated that there was no significant difference (Supplementary Fig. 5D). Consequently, to validate the function of C1q in macrophages, CKO mice were selected for subsequent animal experiments.

Initially, we investigated the impact of C1q deficiency on macrophage antigen presentation in MPE. We conducted experiments to assess the effects of C1q deficiency on CD4^+^ and CD8^+^ T cell proliferation *in vitro*. Macrophages isolated from MPE of C1qa CKO and C1qa^fl/fl^ mice were co-cultured with splenic T cells from OT-I or OT-II mice in the presence of OVA peptides. The results demonstrated a reduced extent of CD8^+^ T cell proliferation when co-cultured with C1q-deficient macrophages compared with control macrophages; although CD4^+^ T cell proliferation exhibited a similar trend, there was no significant differences between the two groups (Fig. 2H and I). The results from mice receiving MC38 intrapleural injection were consistent with these findings (Fig. 2J and K). Additionally, we compared the CD86 and HLA-DR expression between C1q^+^ and C1q^-^ macrophages from MPE patients, flow cytometry results revealed that C1q^+^ macrophages expressed significantly higher CD86 and HLA-DR levels compared to C1q^-^ macrophages (Fig. 2L and M). These findings demonstrate that C1q^+^ macrophages possess a more pronounced antigen presentation ability.

Furthermore, consistent with the pathway enrichment analysis, pleural tumor sections from C1q-deficient mice exhibited diminished vascularization, characterized by fewer and shorter blood vessels compared to the control group (Supplementary Fig. 5E). Additionally, the impact of C1q on macrophage phagocytosis and apoptosis in MPE was evaluated. Despite previous studies demonstrating C1q's involvement in microglial phagocytosis and apoptotic cell clearance 35, surprisingly, the results indicated no significant alterations in macrophage phagocytosis and apoptosis following C1q KO in MPE (Supplementary Fig. 6).

### C1q deficiency in macrophages regulated M2 polarization

**Figure 3:**
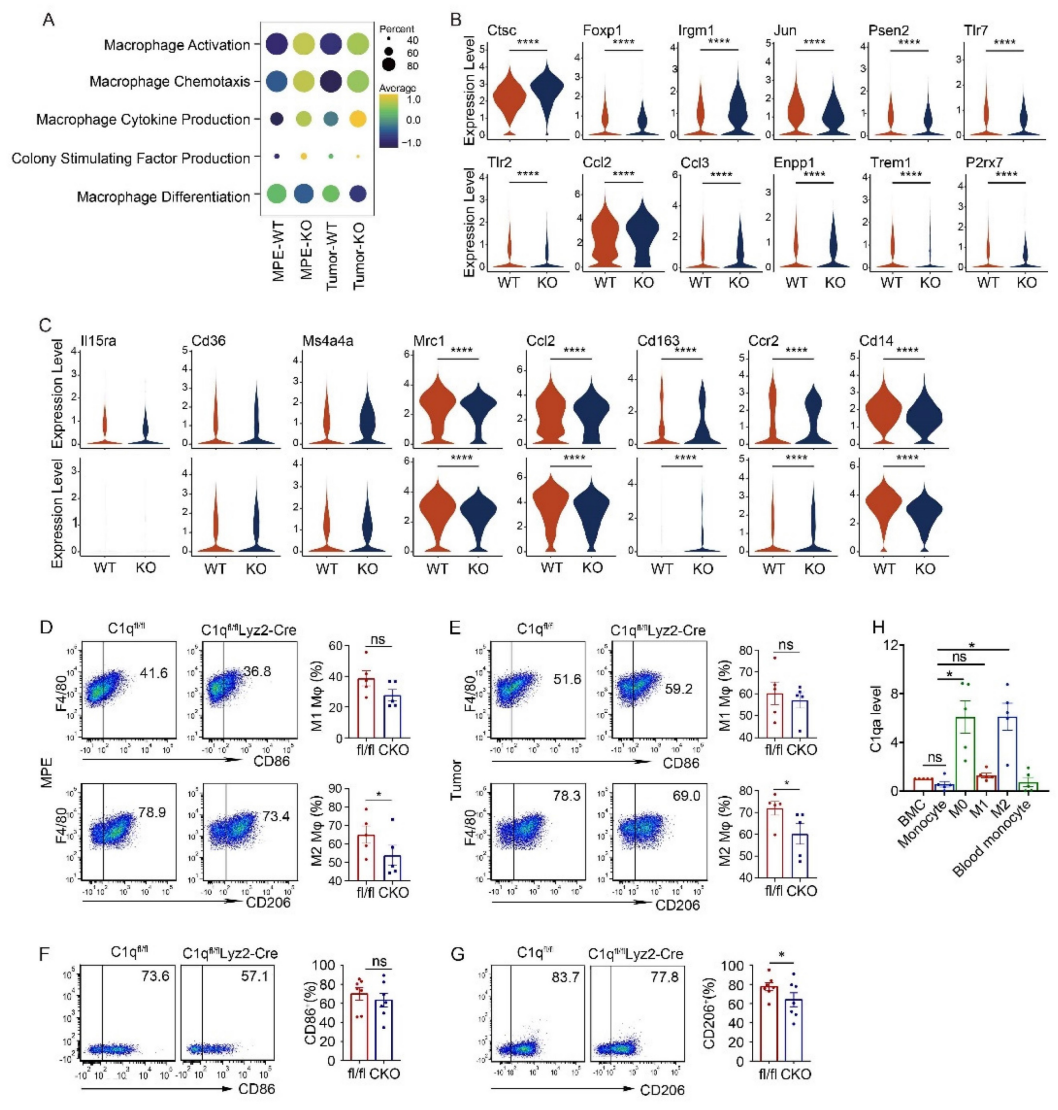
** C1q deficiency in macrophages regulated M2 polarization.** (A) A dot plot showed the level of macrophage activation, chemotaxis, cytokine production, colony-stimulating factor production function and differentiation in the corresponding group. (B) A violin plot showed the expression of corresponding genes in macrophages of the indicated mice. (C) A violin plot showed the expressions of M1-like or M2-like markers in macrophages from the MPE (upper panel) or tumor (bottom panel) of the indicated mice. (D) The representative flow cytometric dot plots (left panel) and statistical comparisons (right panel) of M1 and M2 macrophages in MPE (n = 5). (E) The representative flow cytometric dot plots (left panel) and statistical comparisons (right panel) of M1 and M2 macrophages in pleural tumors (n = 5). (F) The representative flow cytometric dot plots (left panels) and comparisons (right panels) of differentiation of M1 before and after C1q knockdown (n = 7). (G) The representative flow cytometric dot plots (left panels) and comparisons (right panels) of differentiation of M2 before and after C1q knockdown (n = 7). (H) mRNA expression of C1qa in cells at different stages of bone marrow derived macrophage (BMDM) differentiation was quantified by quantitative RT-PCR (n = 5). *P < 0.05, ****P < 0.0001, determined by Student's *t*-test or one-way ANOVA followed by Bonferroni test.

In the C1qa^-/-^ group, macrophage activation, chemotaxis, cytokine production, and colony-stimulating factor production signatures were upregulated, along with associated genes such as Jun, Psen2, Tlr7, Tlr2 Trem1, and P2rx7, while the macrophage differentiation signature was downregulated (Fig. 3A and B). Macrophage polarization reflects the functional differentiation of these cells. Macrophages are categorized into M1 subpopulations, which exhibit proinflammatory functions, and M2 subpopulations, which exhibit anti-inflammatory functions. Within the tumor environment, tumor-associated macrophages tend to be M2, acting as pro-tumor agents 36. In C1qa^-/-^ mice, the expression of M2 macrophage polarization-related genes, like Mrc1, Ccl2, Cd163, Ccr2, and Cd14 decreased, while no significant change was detected in the expression of M1 polarization-related genes (Fig. 3C). Flow cytometry also revealed no difference in the M1 macrophage ratio between C1qa CKO and C1qa^fl/fl^ mouse MPE, with fewer M2 macrophages found in C1qa CKO moue MPE (Fig. 3D). Similar results were observed in macrophages in pleural tumors (Fig. 3E). To investigate the role of C1q in macrophage polarization, bone marrow (BM) cells were isolated from C1qa CKO and C1qa^fl/fl^ mice and cultured under M1 or M2 polarization conditions, respectively (Supplementary Fig. 7A). Consistent with our hypothesis, the results demonstrated that C1q deficiency significantly decreased M2 polarization but appeared to have little effect on M1 polarization (Fig. 3F and G). The mRNA expression level of C1q was also examined at different stages during macrophage polarization (Supplementary Fig. 7A). The results indicated that C1q expression was significantly upregulated at the M0 stage compared to BM cells or monocytes, Interestingly, polarized M2 macrophages expressed relatively higher levels of C1q, while polarized M1 macrophages expressed relatively lower levels of C1q (Fig. 3H, Supplementary Fig. 7B). Collectively, our data suggest that M2 polarization is C1q-dependent, while M1 polarization appears to be independent of C1q. These findings support the notion that C1q affects M2 polarization and macrophage antigen presentation functions in MPE.

### C1q only affected the antigen-presenting phenotype of immature macrophages

**Figure 4:**
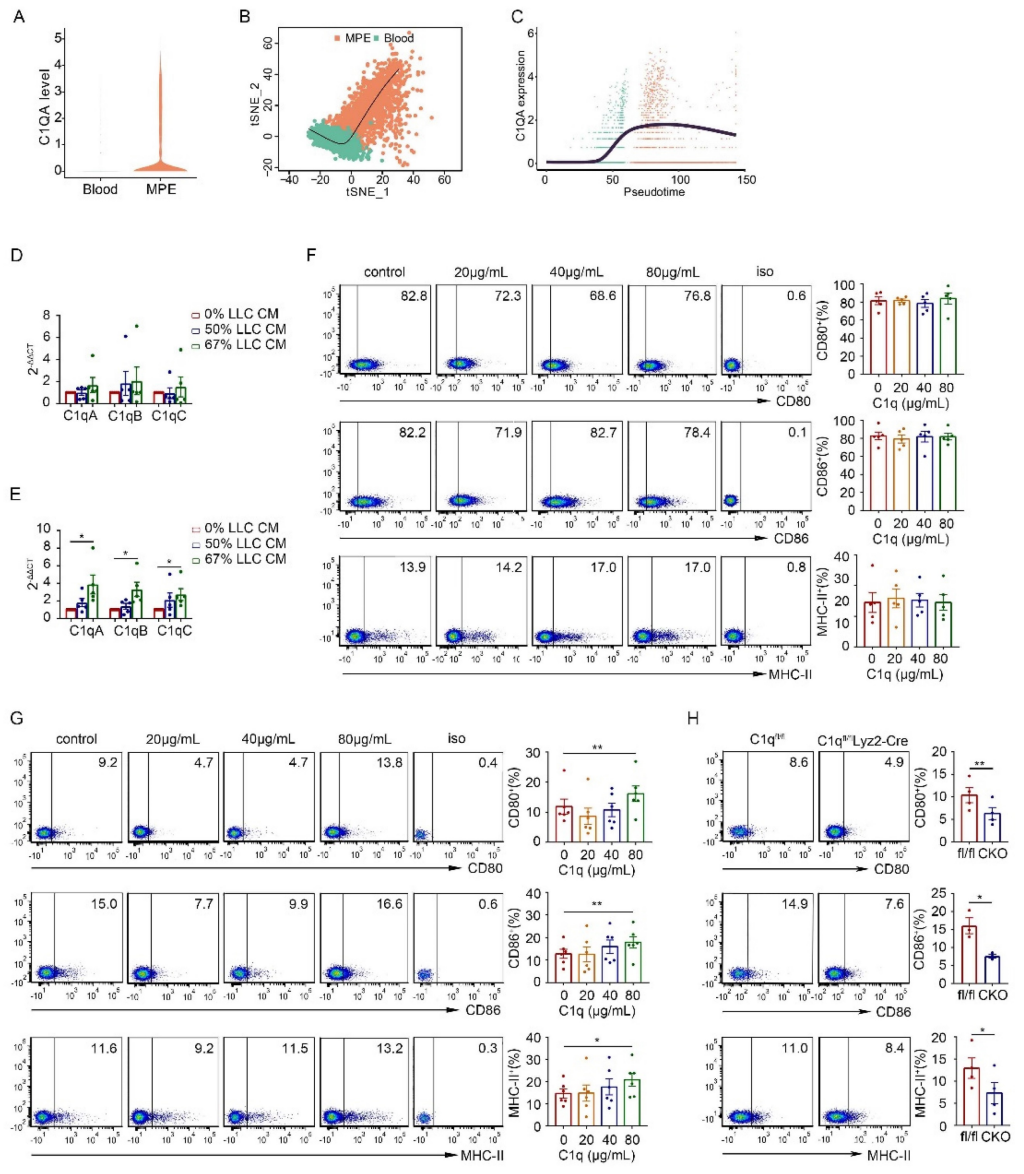
** C1q only affects the antigen-presenting phenotype of immature macrophages.** (A) A violin plot showing the expression of C1QA in monocytes and macrophages from the human MPE and blood scRNA-seq data. (B) Slingshot differentiation trajectory analyses of monocyte and macrophages from the human MPE and blood scRNA-seq data. (C) Slingshot differentiation trajectory analyses of C1QA expression in monocytes and macrophages. (D, E) Macrophages from the normal pleural cavity (D) and BMDM (E) were isolated from WT mice and incubated with LLC conditional medium (CM). The mRNA level of C1q was measured after one day by quantitative RT-PCR (each n = 5). (F, G) Macrophages from the normal pleural cavity (F) and BMDM (G) were isolated from WT mice and incubated with purified C1q. The levels of CD80, CD86 and MHC II were measured after 3 days by flow cytometry (each n = 5). (H) The representative flow cytometric dot plots (left panels) and comparisons (right panels) of CD80, CD86 and MHC II of BMDM before and after C1q knockdown (each n = 3 or 4). Data were presented as means ± SEM. *P < 0.05, **P < 0.01, determined by Student's *t*-test or one-way ANOVA followed by Bonferroni test.

As C1q expression exhibited dynamic changes during macrophage polarization, we analyzed C1q expression in macrophages and peripheral blood monocytes in MPE and its association with the macrophage developmental trajectory in the scRNA-seq data of MPE patients. C1QA expression was significantly higher in MPE macrophages compared to peripheral blood monocytes (Fig. 4A). As monocytes differentiated into macrophages, C1QA expression rapidly increased rapidly and remained elevated in mature macrophages (Fig. 4B and C). Consequently, we hypothesized that C1q plays a more pronounced role in the immature macrophages rather than in mature macrophages.

We further investigated whether tumor cells or the tumor environment influenced the expression of C1q and antigen presentation-related markers on mature macrophages. Initially, we co-cultured macrophages from non-MPE with A549 or BAEAS-2B cell lines. However, regardless of whether macrophages were directly co-cultured with these cells or cultured with their conditional medium (CM), no statistically significant differences were detected in C1q expression levels and antigen presentation-related markers on the macrophages (Supplementary Fig. 7C-E). Addtionally, we tested whether the addition of C1q to the culture affected the expression of antigen presentation-related markers on macrophages. We found that the addition of purified C1q protein to macrophages isolated from non-MPE patients did not alter the expression levels of markers related to antigen presentation (Supplementary Fig. 7F and G). These data suggest that the tumor environment does not affect C1q expression on mature macrophages isolated from PE patients, and the addition of C1q does not influence the expression levels of antigen presentation-related markers on these macrophages. Subsequently, we isolated macrophages from the pleural cavity or BM cells from the femur and tibia of naïve mice and co-cultured them with LLC CM. The mRNA expression of C1q was measured by qPCR one day later. The results suggested that LLC CM did not alter C1q expression in pleural macrophages but significantly upregulated C1q expression in BM cell at approximately 67% concentration within the cultured medium (Fig. 4D and E). These findings indicate that while the tumor environment does not affect C1q expression on mature macrophages isolated from the pleural cavity, it can enhance C1q expression on BM cells during macrophage polarization. Given that macrophages from the PE environment are populations in a relatively mature and terminal polarization state, we hypothesized that C1q might alter the expression of antigen presentation-related markers on immature macrophages. To verify this hypothesis, C1q was added to cultured mouse pleural lavage macrophages and BM cells. Similar to the results from patients (Supplementary Fig. 7F and G), we found that the addition of different concentrations of purified C1q protein had no significant effect on the antigen presentation-related markers of pleural macrophages from mice (Fig. 4F). Interestingly, the CD80, CD86, and major histocompatibility complex class II (MHC-II) levels in BM cells were all increased when the purified C1q protein concentration was 80 μg/mL (Fig. 4G). Moreover, after C1q knockdown, the CD80, CD86, and MHC-II levels decreased (Fig. 4H). Collectively, these data suggest that C1q expression is significantly upregulated at the M0 stage compared to BM cells or monocytes, and M2 polarization is C1q-dependent. The addition of C1q affects the antigen presentation of cells in a relatively immature state, while C1q deficiency affects the antigen presentation of mature macrophages with high C1q expression.

### C1q deficiency in macrophages rescued exhausted phenotype of CD8^+^ T cells in MPE

**Figure 5:**
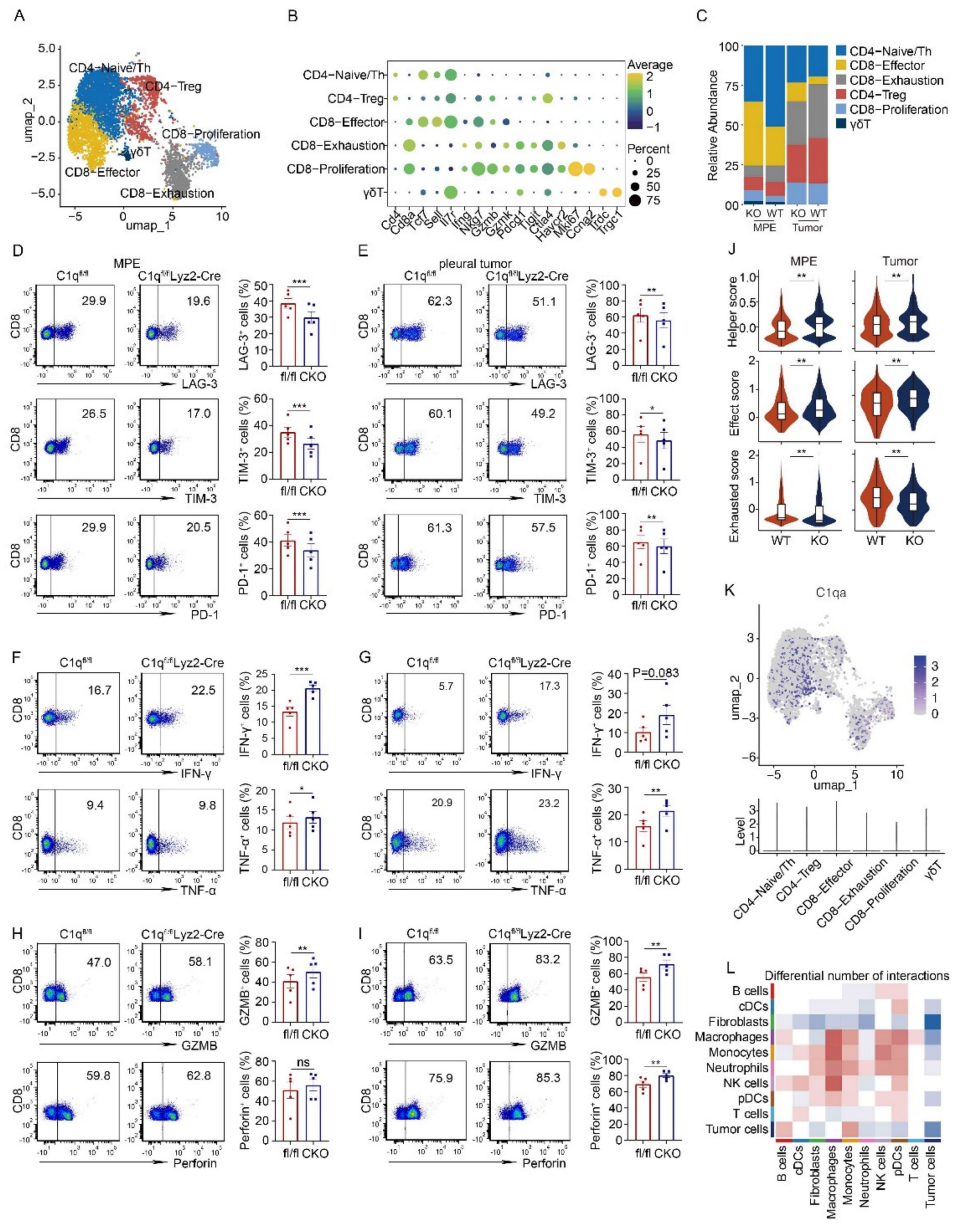
** C1q deficiency in macrophages rescued the exhausted phenotype of CD8^+^ T cells in MPE.** (A) UMAP plot of the T cells in the MPE and tumor tissue from WT mice or C1qa^-/-^ mice. (B) Dot plot showed the expression of the marker genes in the corresponding cell cluster. (C) Bar graphs showed the proportion of each cell cluster. (D) The representative dot plots from flow cytometric analysis (left panel) and statistical comparisons (right panel) of LAG-3^+^, TIM-3^+^, PD-1^+^ exhausted CD8^+^ T cells in MPE between C1q^fl/fl^ and C1q^fl/fl^Lyz2-Cre mice receiving intrapleural injection of LLC cells (each n = 5). (E) The representative dot plots from flow cytometric analysis (left panel) and statistical comparisons (right panel) of LAG-3^+^, TIM-3^+^, PD-1^+^ exhausted CD8^+^ T cells in pleural tumors between C1q^fl/fl^ and C1q^fl/fl^Lyz2-Cre mice receiving intrapleural injection of LLC cells (each n = 5). (F) The representative dot plots from flow cytometric analysis (left panel) and statistical comparisons (right panel) of IFN-γ^+^, TNF-α^+^ CD8^+^ T cells in MPE between C1q^fl/fl^ and C1q^fl/fl^Lyz2-Cre mice receiving intrapleural injection of LLC cells (each n = 5). (G) The representative dot plots from flow cytometric analysis (left panel) and statistical comparisons (right panel) of IFN-γ^+^, TNF-α^+^ CD8^+^ T cells in pleural tumors between C1q^fl/fl^ and C1q^fl/fl^Lyz2-Cre mice receiving intrapleural injection of LLC cells (each n = 5). (H) The representative flow cytometric dot plots (left panel) and statistical comparisons (right panel) of GZMB^+^, Perforin^+^ CD8^+^ T cells in MPE between C1q^fl/fl^ and C1q^fl/fl^Lyz2-Cre mice receiving intrapleural injection of LLC cells (each n = 5). (I) The representative flow cytometric dot plots (left panel) and statistical comparisons (right panel) of GZMB^+^, Perforin^+^ CD8^+^ T cells in pleural tumors between C1q^fl/fl^ and C1q^fl/fl^Lyz2-Cre mice receiving intrapleural injection of LLC cells (each n = 5). (J) A violin plot showed the expression of helper score in CD4^+^ T cells (upper panel) and effect and exhausted scores in CD8^+^ T cells (middle and bottom panel) from the MPE or tumor of the indicated mice. (K) UMAP plot (upper panel) and violin plot (bottom panel) of C1qa. (L) A heatmap showed the number of the cell-to-cell interactions between the corresponding cell clusters. Data were presented as means ± SEM. *P < 0.05, **P < 0.01, ***P < 0.001; the statistical comparisons were determined by Student's *t*-test.

T lymphocytes constitute a significant cell population in MPE. According to the expression of classical marker genes, T cells were categorized into CD4-Naïve/Th cells, CD4-Treg cells, CD8-Effector cells, CD8-Exhaustion, CD8-Proliferation, and γδT cells (Fig. 5A and B). In C1qa^-/-^ mice, the CD8-Effector cell proportion increased, while the CD8-Exhaustion cell proportion decreased. Flow cytometry also revealed a downregulation in exhausted CD8^+^ T cells, as evidenced by the presence of fewer LAG-3, PD-1, and TIM-3-positive CD8^+^ T cells in the MPE of C1qa CKO mice compared to C1qa^fl/fl^ mice, suggesting that C1q^+^ macrophages promoted an exhausted state in CD8^+^ T cell within the MPE. The pleural tumors exhibited a similar trend (Fig. 5C-E). Furthermore, C1q deficiency in macrophages significantly increased the expression of effector and cytotoxic molecules like IFN-γ, TNF-α and GZMB, although no statistical difference was detected for perforin expression between the two groups (Fig. 5F and G). In the pleural tumors, C1q deficiency in macrophages significantly increased TNF-α, GZMB and perforin expression, although IFN-γ showed an upward trend in the C1q-deficient group, there was no statistically significant difference (Fig. 5H and I). Flow cytometry also unveiled that Th1 and Th17 cells were significantly upregulated, while Treg cells were downregulated in the MPE of C1qa CKO mice compared to C1qa^fl/fl^ mice (Supplementary Fig. 8), which was not evident in the scRNA-seq data due to the small sample size. The scRNA-seq data also demonstrated that CD4^+^ T cells from C1qa^-/-^ mice had a phenotype with enhanced helper-like features, while CD8^+^ T cells displayed a phenotype with increased effector-like characteristics and decreased exhaustion in both MPE and pleural tumor cells (Fig. 5J). As C1qa expression was hardly detected in all T cell subclusters (Fig. 5K), we hypothesized that the interaction between cell ligand-receptor pairs might play a vital role in regulating T cells. CellChat analysis revealed that compared with the C1qa^-/-^ mice, only macrophages in WT mice exhibited increased ligand-receptor interactions with T cells, indicating that macrophages were the predominant cell type regulating T cell phenotype in the MPE microenvironment (Fig. 5L). In summary, our findings demonstrate that C1q deficiency in macrophages can suppress MPE development through increasing the number of functional effector T lymphocytes while decreasing the number of tumor-associated macrophages and inhibitory and exhausted T lymphocytes.

### C1q deficiency in macrophages increased NK cell activity in MPE

**Figure 6:**
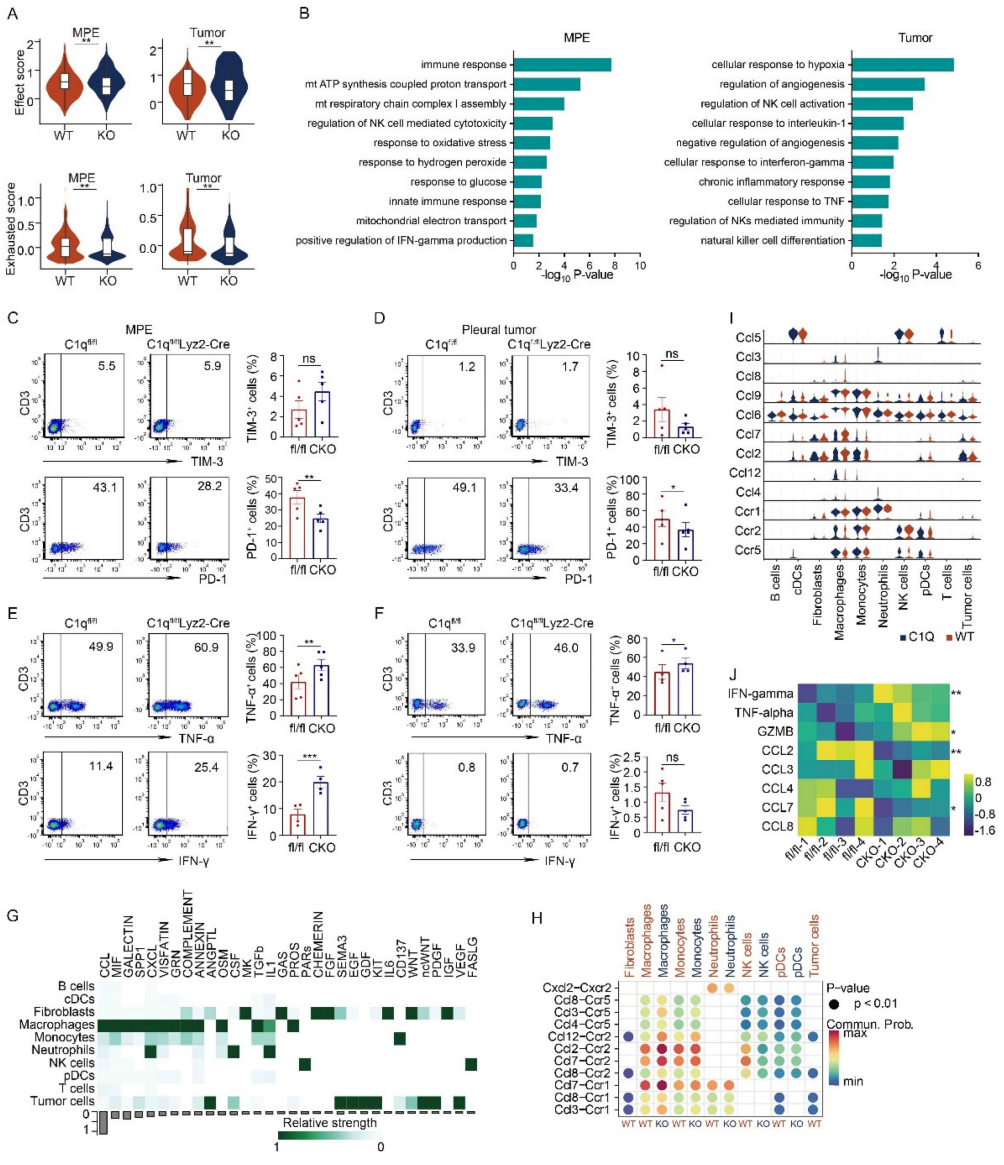
** C1q deficiency in macrophages increased NK cell activity in MPE.** (A) A violin plot showed the effect and exhausted scores in NK cells from the MPE or tumor of the indicated mice. (B) A bar graph showed the enrichment pathways of the upregulated genes in NK cells of MPE (left panel) or tumor (right panel) between C1qa^-/-^ and WT mice. (C) The representative dot plots from flow cytometric analysis (left panel) and statistical comparisons (right panel) of TIM-3^+^, PD-1^+^ NK cells in MPE between C1q^fl/fl^ and C1q^fl/fl^Lyz2-Cre mice receiving intrapleural injection of LLC cells (each n = 5). (D) The representative dot plots from flow cytometric analysis (left panel) and statistical comparisons (right panel) of TIM-3^+^, PD-1^+^ NK cells in pleural tumors between C1q^fl/fl^ and C1q^fl/fl^Lyz2-Cre mice receiving intrapleural injection of LLC cells (each n = 5). (E) The representative dot plots from flow cytometric analysis (left panel) and statistical comparisons (right panel) of IFN-γ^+^, TNF-α^+^ NK cells in MPE between C1q^fl/fl^ and C1q^fl/fl^Lyz2-Cre mice receiving intrapleural injection of LLC cells (each n = 5). (F) The representative dot plots from flow cytometric analysis (left panel) and statistical comparisons (right panel) of IFN-γ^+^, TNF-α^+^ NK cells in pleural tumors between C1q^fl/fl^ and C1q^fl/fl^Lyz2-Cre mice receiving intrapleural injection of LLC cells (each n = 5). (G) A heatmap showed the cell-to-cell interactions strength of each pathway in the corresponding cell cluster. (H) A dot plot showed the cell-to-cell communication probability of each ligand-receptor pair in the corresponding cell cluster. (I) A violin plot indicated the expression of the ligand genes and receptor genes in the chemoattractant cytokine ligand (CCL) pathway in the corresponding cell cluster. (J) Heatmap showed the expression of IFN γ, TNF α, GZMB, CCL2, CCL3, CCL4, CCL7 and CCL8 in the MPE of the indicated mice detected by Luminex Assays. Data were presented as means ± SEM. *P < 0.05, **P < 0.01, ***P < 0.001; the statistical comparisons were determined by Student's *t*-test.

NK cells constitute a significant portion of innate immune cells in MPE and function in MPE development. Compared to LAG-3 and TIM-3, PD-1 exhibited substantially higher expression in NK cells, functioning as an inhibitory molecule. In C1qa^-/-^ mice, NK cells displayed a phenotype characterized by increased effector-like features and decreased exhausted features in both MPE and pleural tumor cells. NK cell activation and immune response were enriched in C1qa^-/-^ mice (Fig. 6 A and B). Flow cytometry analysis further revealed that C1q deficiency in macrophages significantly reduced the expression of the inhibitory molecule PD-1 in NK cells, while no statistical difference was observed for TIM-3 between the two groups (Fig. 6C and D). Moreover, we compared the effector molecule expression on NK cells in MPE from C1qa^fl/fl^ and C1qa CKO mice. The results demonstrated that C1q deficiency in macrophages significantly upregulated IFN-γ and TNF-α expression (Fig. 6E and F). Interestingly, in contrast to those in MPE, NK cells in pleural tumors expressed remarkably low levels of IFN-γ (Fig. 6F).

Cell-to-cell interaction analysis indicated that among all the different signaling pathways between the C1qa^-/-^ and WT mice, the CCL pathway exhibited the highest relative strength and was primarily contributed by macrophages (Fig. 6G). Analysis of ligand-receptor pairs in the CCL pathway sourced by macrophages with significant differences between the C1qa^-/-^ and WT mice demonstrated that the communication probability of Ccl2-Ccr2, Ccl7-Ccr2, and Ccl8-Ccr2 was decreased in NK cells from C1qa^-/-^ mice (Fig. 6H). Across all cell types, Ccr2 expression was higher in NK cells and lower in the C1qa^-/-^ mice compared to the WT ones. The expressions of Ccl2 and Ccl7 in macrophages was lower in the C1qa^-/-^ mice than in the WT ones (Fig. 6I), and Luminex analysis demonstrated that the CCL2 and CCL7 levels in the pleural effusion of CKO mice were significantly lower than those of C1qa^fl/fl^ mice (Fig. 6J). These findings indicate that the CCR2 receptor on NK cells functions in the regulation of NK cells by macrophages.

### CCR2 inhibitor and hippuric acid inhibited MPE formation in mice

**Figure 7:**
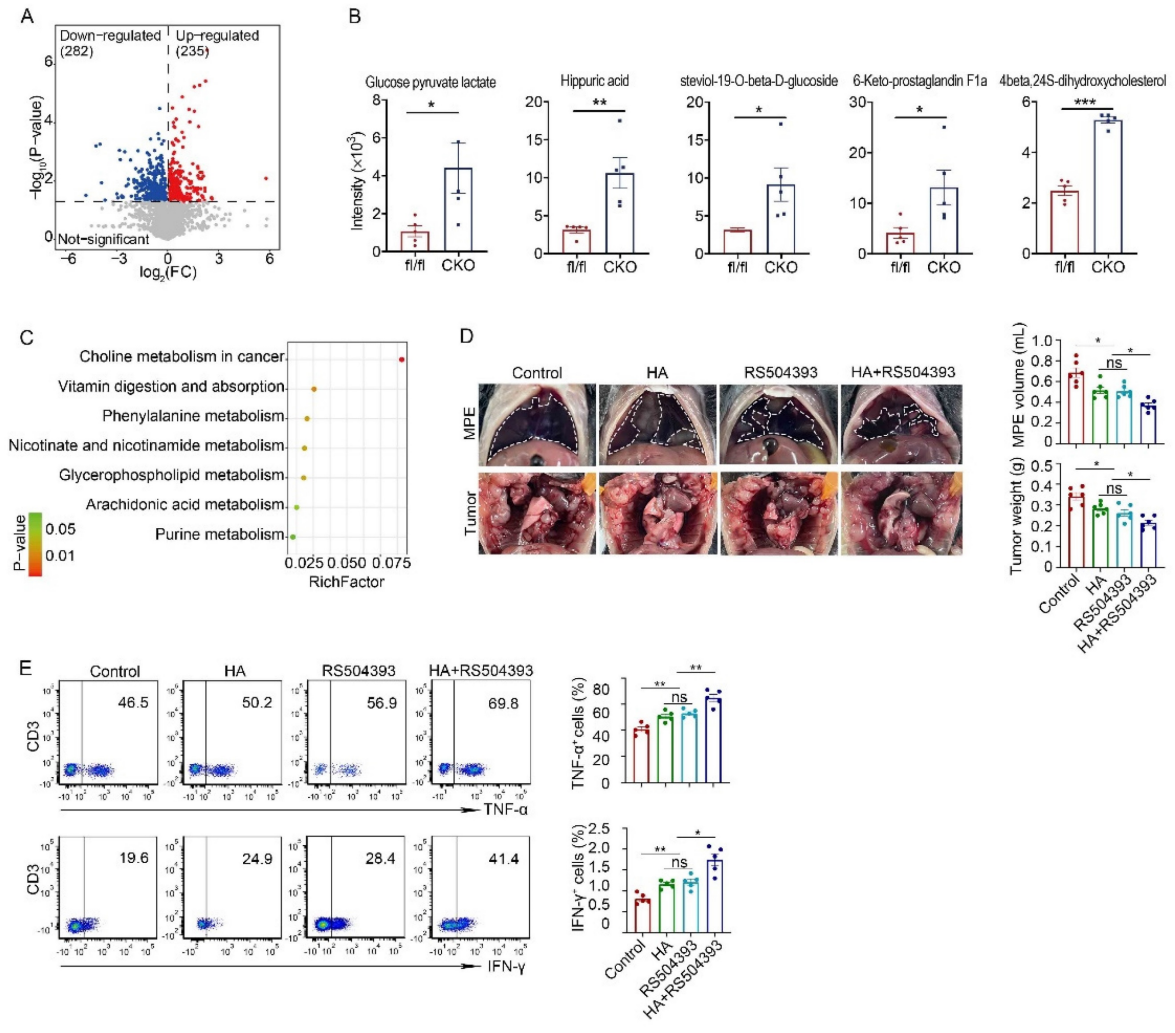
** CCR2 inhibitor and hippuric acid inhibited MPE formation in mice.** (A) A volcano plot showed 235 up-regulated metabolites and 282 down-regulated metabolites (P < 0.05, variable important in projection (VIP) > 1) in MPE between C1q^fl/fl^ and C1q^fl/fl^Lyz2-Cre mice. (B) A box plot of the metabolic intensity of the corresponding metabolite. (C) Pathway enrichment analysis of the differential metabolites. (D) Anatomical images of pleural effusion and tumors of MPE mice receiving hippuric acid and/or RS504393 (left panels). Comparisons of MPE volume (each n = 5) and pleural tumor mass (each n = 5) among different treatment groups (right panels). (E) The representative dot plots from flow cytometric analysis (left panels) and statistical comparisons (right panels) of TNF-α^+^ or IFN-γ^+^ NK cells in MPE among different treatment groups. Data were presented as means ± SEM. *P < 0.05, **P < 0.01, ***P < 0.001 determined by Student's *t*-test or one-way ANOVA followed by Bonferroni test.

Metabolic reprogramming in the microenvironment also serves as a significant regulatory factor of immune cell functional status. To investigate whether C1q KO affected the metabolic microenvironment of MPE, we performed a metabolomic assay of MPE in CKO and C1qa^fl/fl^ mice. A comprehensive list of 4182 metabolites was identified, encompassing amino acids, fatty acids, carbohydrates, and various other compounds. Orthogonal Partial Least Squares Discrimination Analysis (OPLS-DA) of the metabolite profiles effectively distinguished each mouse group, indicating significant variations in the types and concentrations of metabolites among the diverse treatment groups of mice (Supplementary Fig. 9A and B). A total of 518 differential metabolites were detected in the MPE of mice between CKO and C1qa^fl/fl^ mice, with 235 metabolites upregulated and 283 metabolites downregulated in the MPE of CKO mice (Fig. 7A). In order of variable important in projection (VIP) ranking, the metabolites that exhibited the most significant elevation in MPE of CKO mice were glucose pyruvate lactate, hippuric acid, steviol-19-O-beta-D-glucoside, 6-Keto-prostaglandin F1a, and 4beta,24S-dihydroxycholesterol (Fig. 7B). We conducted the Kyoto Encyclopedia of Genes and Genomes (KEGG) analysis to assess the differentially expressed metabolites between the two groups. The first three enriched metabolic pathways were choline metabolism in cancer, vitamin digestion and absorption, phenylalanine metabolism and nicotinate and nicotinamide metabolism (Fig. 7C). Our ELISA results also demonstrated that compared with C1qa^fl/fl^ mice, the level of hippuric acid in MPE of CKO mice was significantly increased, indicating that hippuric acid might have an inhibitory effect on MPE (Supplementary Fig. 9C). To verify whether hippuric acid treatment was associated with macrophage phenotype, we carried out related experiments with macrophages isolated from mouse pleural cavity and bone marrow. Consistent with the results from C1q protein treatment (Fig. 4F and G), we found that treatment of hippuric acid did not affect the expression of antigen presentation-related molecules on macrophages isolated from the pleural cavity; conversely, treatment of hippuric acid significantly upregulated the CD80 and MHC-II expression on bone marrow-derived macrophage (BMDM) (Supplementary Fig. 10A and B). More interestingly, our data also demonstrated that treatment of hippuric acid decreased M2 macrophage polarization (Supplementary Fig. 10C), consistent with the results from C1q deficient mice.

Research has demonstrated the potential of hippuric acid therapy in regulating glucose homeostasis in mice. Additionally, RS504393, a highly selective CCR2 inhibitor, has shown promise in treating ischemia-reperfusion injury in murine models. Consequently, we tested the therapeutic effect of hippuric acid and CCR2 inhibition in MPE models. To assess the impact of hippuric acid and RS504393 on MPE, mice were administered these agents individually or in combination. The results indicated that treatment with either hippuric acid or RS504393 alone inhibited MPE and tumor growth, while the combination of both agents exhibited a more pronounced inhibitory effect (Fig. 7D). Furthermore, we examined the TNF-α and IFN-γ expressions in NK cells following treatment with hippuric acid and/or RS504393. As anticipated, treatment with either hippuric acid or RS504393 individually enhanced TNF-α and IFN-γ expression in NK cells, while the combination of both agents resulted in a more substantial increase in the cytokine expression (Fig. 7E).

## Discussion

Complement C1q is a crucial factor that regulates immune responses on both of innate and adaptive aspects and is implicated in various of diseases 37. However, the function and mechanism of C1q in cancers and cancer-related diseases remain controversial. Recently, Zhang *et al.* described the role of C1q in MPE and discovered that fatty acid metabolism mediated by fatty acid binding protein 5 (FABP5) contributed to the immunosuppression of C1q^+^ macrophages in MPE. As mentioned in the introduction, MHC-II/HLA-DR^+^CX3CR1^+^ and MHC-II/HLA-DR^-^CX3CR1^-^ were used to define and isolate "C1q^+^" and "C1q^-^" macrophages, respectively. This approach is more suitable for investigating the functions of these macrophage clusters in MPE, but it may not be optimal for examining C1q-mediated macrophages in MPE.

This study provides a comprehensive picture of the role of C1q^+^ macrophages in modulating immune responses within MPE. Utilizing C1qa KO mice, the data demonstrate that C1q deficiency in macrophages suppresses MPE, inhibits tumor growth, and prolongs murine survival. C1q^+^ macrophages display enhanced antigen presentation capabilities. The absence of C1q in macrophages augments the immune activity and tumor-killing capacity of CD8^+^ T cells and NK cells in the MPE microenvironment through cell-to-cell interactions and metabolic reprogramming of the pleural effusion.

Previous studies have reported that C1q function as a tumor-promoting factor in the tumor microenvironment which is the complement cascade-independent 24**.** The current study demonstrated a significant difference in C1q concentration between MPE and non-MPE supernatants, while C3 and C4 levels remained unchanged. This finding suggests that C1q may also can function in MPE in a complement cascade-independent manner. Our results demonstrate that both global C1q deficiency and macrophage-specific C1q deficiency can reduce MPE volume, inhibit pleural tumor growth, and prolong mouse survival, with no significant survival difference observed between the C1qa^-/-^ and C1qa CKO mice. Supporting this phenomenon, our scRNA-seq and flow cytometry analyses revealed that macrophages were the primary source of C1q in MPE.

Studies have documented that C1q regulates the differentiation of monocytes into macrophages 38, 39. Our study uncovered that C1q expression is upregulated during macrophage maturation from BM cells and influences M2 macrophage polarization. Compared to BM cells, C1q expression was elevated at the M0 stage and further increased in polarized M2 macrophages. Notably, C1q deficiency significantly decreased M2 polarization *in vitro.* Consistent with the results *in vitro*, we found that C1q deficiency significantly reduced the proportion of M2 macrophages in mouse MPE. Decreasing M2 macrophages or "re-educating" pro-cancer M2 macrophages into anti-cancer M1 macrophages has been documented as a promising strategy to enhance cancer therapy 40. Pleural effusion from malignant pleural mesothelioma promoted M2 polarization and the macrophages exhibited an immunosuppressive function within MPE 41. Targeting C1q as a macrophage-based immunotherapy strategy is a promising approach that warrants further investigation.

Lymphocytes accounted for most of the immune cells existing in MPE, Our and others' works have demonstrated that Th1, Th17, Th9, Th22, Treg, CD8^+^ T and B cells were all involved in MPE development 7, 29, 42, 43. In our study on C1q, we found that C1q deficiency in macrophages increased Th1 and Th17 cells, but decreased Treg cells in mouse MPE. These results were consistent with others' studies in systemic lupus erythematosus (SLE) and emphysema, which showed that C1q-polarized macrophages reduced proliferation of Th17 and Th1 subsets, decreased differentiation of Th17 cells, and increased Treg development and proliferation 44, 45. By using C1q-deficient mice, we found that C1q KO in macrophages significantly diminished the exhaustion status of CD8^+^ T cells. The LAG-3, TIM-3 and PD-1 expression on CD8^+^ T cells decreased in C1q-deficient mice compared with control ones, both in MPE and pleural tumors, which is consistent with conclusions derived from using "C1q^+^" and "C1q^-^" macrophages 29. However, we did not find FABP5 to be a differentially expressed gene when comparing C1qa-deficient macrophages with controls.

Cytotoxic T lymphocytes (CTLs) and NK cells are essential elements in the defense against tumor development and represent promising candidates for cancer immunotherapy through two major pathways: granzymes and the TNF superfamily 46-48. Extensive evidence demonstrates that CD8^+^ CTLs and NK cells can identify and destroy tumor cells through the secretion of perforin, granzymes, and cytokines such as interferon-gamma (IFN-γ) and TNF-α 46, 49-52. Although NK cells effectively restrain tumor growth and metastasis, they are scarce in the tumor environment 14. The current study provides evidence that NK cells are present and functional in MPE and that C1q^+^ macrophages contribute to the dysfunction of CD8^+^ T and NK cells.

Studies have demonstrated that C1q promotes DC maturation and influences secretion of cytokines and immune responses of T cells 53. The ligation of CD40, rather than toll-like receptors (TLRs), contributed to C1q-mediated CD80, CD86, and MHC-II expression, together with interleukin-12 (IL-12) production on DCs, ultimately impacting antigen presentation to T cells. The ligation of CD40 complexed with C1q on DCs increased IL-12 production through the upregulation of phosphorylation of extracellular signal-regulated kinase 1/2 (ERK-1/2) and p38 mitogen-activated protein kinases (MAP kinases), consequently enhancing antigen presentation to T cells and IFN-γ production 54. Dong *et al.* recently reported that high Epstein-Barr virus-induced gene 3 (Ebi3) expression levels, regulated by the deficiency of RNA N^6^-adenosine methyltransferase Mettl14 in C1q^+^ macrophages, promoted dysfunction of CD8^+^ T cells and tumor growth 55. In human SLE, C1q collaborates with high mobility group box 1 protein (HMGB1) to promote M2 macrophage polarization from monocytes through the receptor for advanced glycation end products (RAGE) and leukocyte-associated immunoglobulin-like receptor 1 (LAIR-1) in lipid rafts 56. C1q secreted by macrophages ss internalized by CD8^+^ T cells, binding to and regulating mitochondrial metabolism, which contributes to CD8^+^ T cell dysfunction and exhaustion 57. Recent findings suggest that C1q derived from central nervous system (CNS)-resident macrophages, known as microglia, is internalized and accumulates in neurons with age, undergoing liquid-liquid phase separation in the presence of RNA and functioning in intraneuronal interactions in a temporally and spatially regulated manner 58. Future studies should investigate whether the aforementioned mechanisms are involved in C1q-mediated macrophage activity in the pathogenesis of MPE.

Multiple studies have reported that C. *sporogenes*,* Alistipes indistinctus* and* Bifidobacterium* can metabolize compounds to yield hippuric acid, which cross-talks with and regulates host immunity 59-61. A high-salt diet induces an increase in gut *Bifidobacterium* and hippuric acid levels, enhancing NK cell activation in anti-tumor immunity 61. In current study, we observed that hippuric acid levels were elevated in MPE from mice with C1q deficiency in macrophages. Previous studies have suggested that the CCL2-CCR2 signaling axis is a beneficial and promising target for cancer treatment. Cell-to-cell interaction analysis revealed that C1q deficiency could block the CCR signaling axis. RS504393, a selective CCR2 antagonist, can assist and increase NK cell antitumor effects mediated by IL-6 blocking with a neutralizing antibody 60, 62. Treatment with hippuric acid and/or RS504393 can activate NK cell activity and inhibit MPE and tumor growth, with the combination of the two reaching a more pronounced extent. Future studies should focus more on metabolites like hippuric acid mediated by gut microbiota in MPE and cancer treatment, and more efforts should be made to investigate the efficacy of hippuric acid and/or RS504393 treatment in combination with anti-PD-1 drugs for MPE and cancer therapy. Altogether, we provided the direct evidence to interpret the role of C1q-mediated macrophages in the immunosuppression of MPE and offered novel potential strategies for MPE cancer therapy.

## Supplementary Material

Supplementary figures.

## Abbreviations

MPE: malignant pleural effusion
OS: overall survival
scRNA-seq: single-cell mRNA sequencing
TPE: tuberculous pleural effusion
PF: pleural fluid
NSCLC: non-small cell lung carcinoma
DMEM: Dulbecco's modified Eagle's medium
ICs: immune complexes
ELISA: Enzyme-Linked Immunosorbent Assay
BMDM: Murine bone marrow-derived macrophage
KO: knockout
CKO: conditional knockout
CM: conditional medium
TCGA: The Cancer Genome Atlas
LUAD: Lung Adenocarcinoma
