# Supplementary material for: Complement C1q is a key player in tumor-associated macrophage-mediated CD8+ T cell and NK cell dysfunction in malignant pleural effusion
Source: Int J Biol Sci. 2024 Nov 4;20(15):5979–98. doi: 10.7150/ijbs.100607 (PMC11628339; doi:10.7150/ijbs.100607)
Supplement: Supplementary file 1 — Supplementary figures. [file ijbsv20p5979s1.pdf]

Supplementary Figure 1

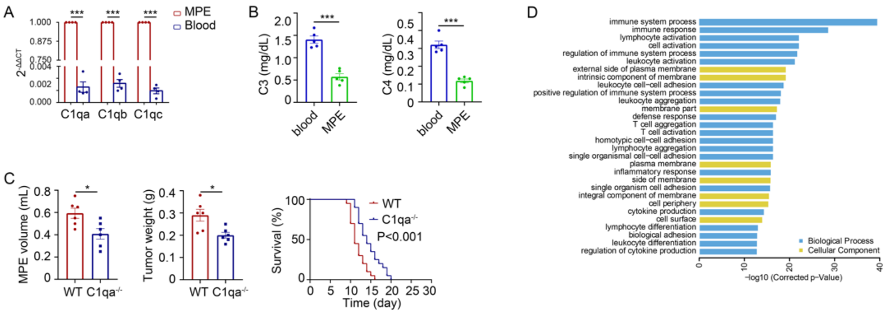

Supplementary Figure 1. C1q was involved in malignant pleural effusion (MPE) development. (A) mRNA expression of C1q in nucleated cells from mouse MPE and blood was quantified by quantitative RT-PCR (n = 4). (B) Comparisons of C3 and C4 concentrations in MPE and paired blood from MPE patients. (C) Comparisons of MPE volume (each n = 6) and pleural tumor mass (each n = 6) between wild-type (WT) and C1qa$^{-/-}$ mice receiving intrapleural injection of MC38 cells. (D) A bar graph showed the enrichment pathways of the different expression genes from macrophages in MPE between C1qa$^{-/-}$ and WT mice by bulk RNA sequencing. Data were presented as means ± SEM. *P < 0.05, ***P < 0.001, determined by Student's *t*-test or pairwise log-rank test (survival analysis).

Supplementary Figure 2

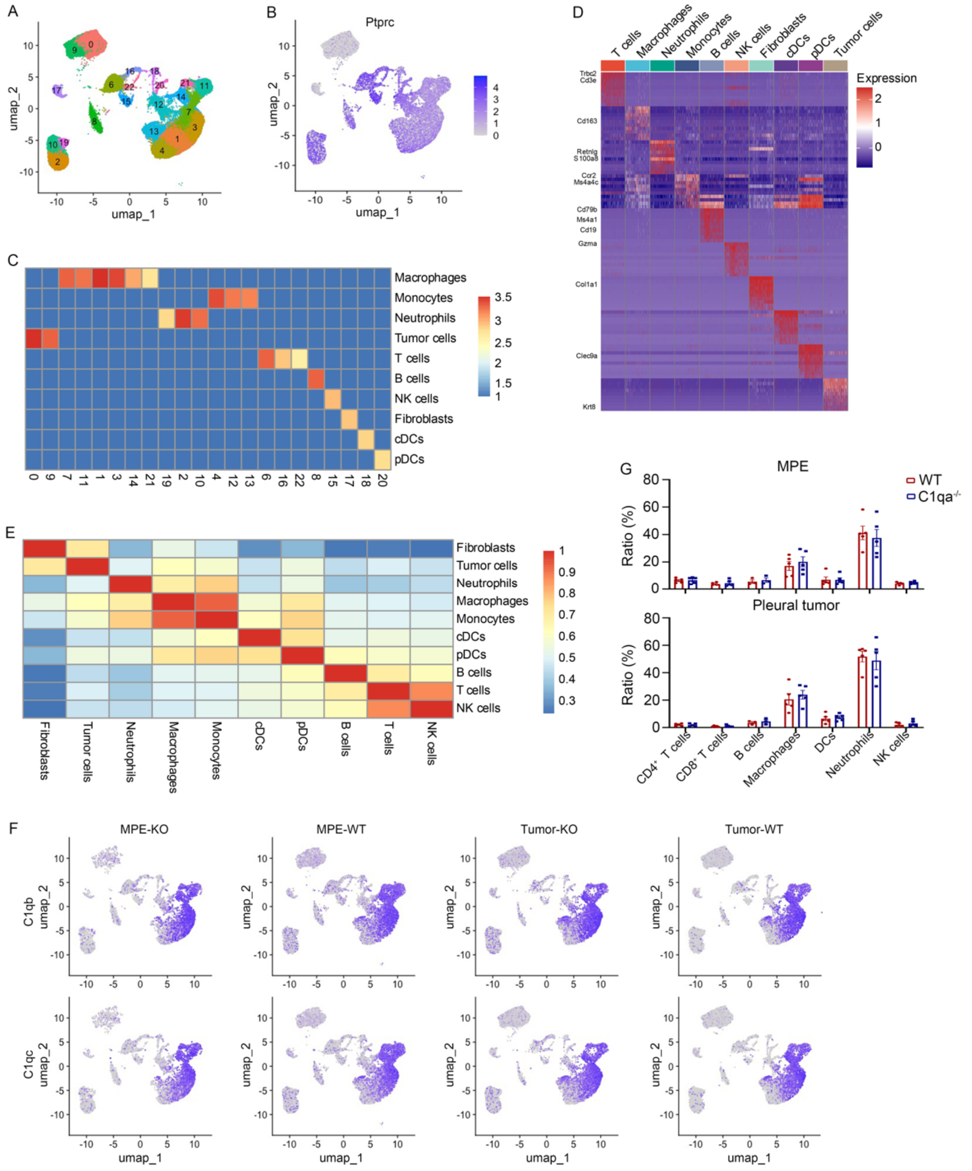

Supplementary Figure 2. Single cell RNA sequencing of cells in MPE mouse model.

(A) UMAP plot of the scRNA-seq data of the pleural effusion and tumor tissue from WT mice or C1qa$^{-/-}$ mice. (B) The expression of Ptprc was shown by a color gradient in the UMAP plot. (C) A heatmap showed the enrichment score of the classical cell type in the corresponding cell clusters. (D) A heatmap showed the expression of the marker genes in the corresponding cell cluster. (E) A heatmap showed the correlation between the corresponding cell clusters. (F) The expression of C1qb and C1qc was shown by a color gradient in the UMAP plot. (G) Comparisons of different cell type compositions in MPE and pleural tumor between WT and C1qa$^{-/-}$ mice receiving intrapleural injection of LLC cells. Data were presented as means ± SEM, determined by Student's *t*-test.

Supplementary Figure 3

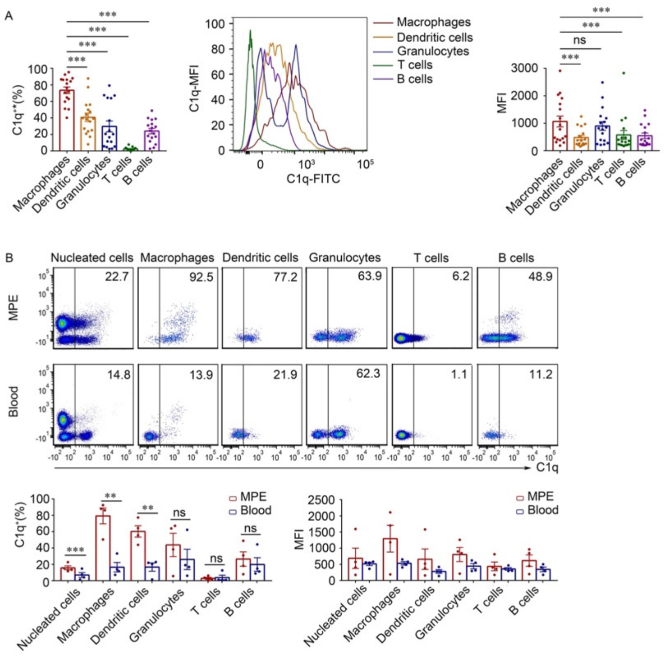

Supplementary Figure 3. C1qa expression in immune cell subsets.

(A) The statistical comparisons of C1q level among nucleated cells from patients' MPE determined by flow cytometry (left panel), the representative flow cytometric MFI plot (middle panel) and statistical comparisons (right panels) C1q level in nucleated cells in patients' MPE (n = 17). (B) The representative flow cytometric dot plots (upper panels) and statistical comparisons (lower panels) of C1q level in nucleated cells between patients' MPE and blood (n = 4). Data were presented as means ± SEM. **P < 0.01, ***P < 0.001, determined by Student's *t*-test (B), or one-way ANOVA followed by Bonferroni test (A)**.**

Supplementary Figure 4

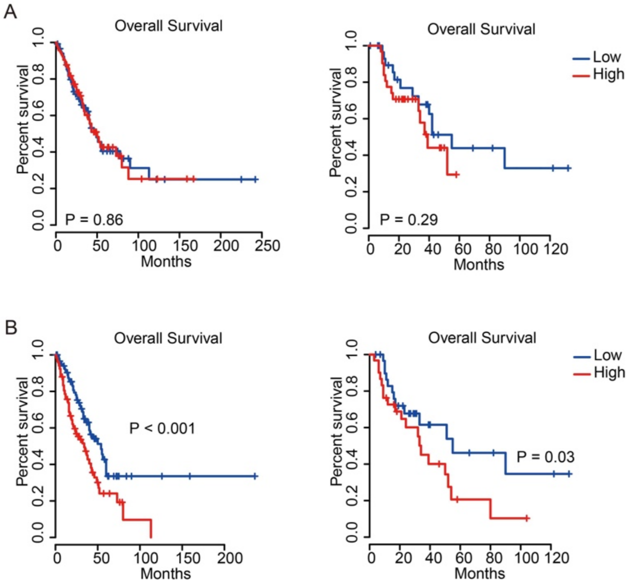

Supplementary Figure 4. Survival analysis for C1qa in The Cancer Genome Atlas (TCGA) Lung Adenocarcinoma (LUAD) data.

(A) The Kaplan-Meier overall survival curves of TCGA LUAD patients (left panel) or TCGA LUAD patients with stage III and IV (right panel) grouped by C1qa expression. The high and low group are divided by the median value of mean expression of C1qa. (B) The Kaplan-Meier overall survival curves of TCGA LUAD patients (left panel) or TCGA LUAD patients with stage III and IV (right panel) grouped by C1qa expression. The high and low group are divided by the median value of mean expression of C1qa after normalization by the proportion of macrophages calculated by CIBERSORT.

Supplementary Figure 5

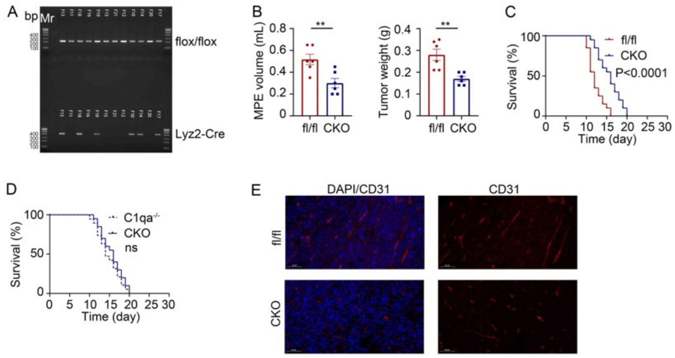

Supplementary Figure 5. Macrophage-specific Cq1a knockout mice were constructed.

(A) Genotyping of C1qa$^{-/-}$ and C1q$^{fl/fl}$Lyz2-Cre mice. (B) Comparisons of MPE volume (each n = 6) and pleural tumor mass (each n = 6) between C1q$^{fl/fl}$ and C1q$^{fl/fl}$Lyz2-Cre mice receiving intrapleural injection of MC38 cells. (C) Life span analysis was performed in C1q$^{fl/fl}$ and C1q$^{fl/fl}$Lyz2-Cre mice receiving intrapleural injection of MC38 cells. Overall survival of the two groups was analyzed using the Kaplan–Meier method and a pairwise log-rank test (each n = 20). (D) Life span analysis was performed in C1qa$^{-/-}$ and C1q$^{fl/fl}$Lyz2-Cre mice receiving intrapleural injection of MC38 cells. Overall survival of the two groups was analyzed using the Kaplan–Meier method and a pairwise log-rank test (each n = 20). (E) Pleural tumor sections were stained with immunofluorescent anti-CD31 mAb to identify the tumor vessels. Data were presented as means ± SEM. **P < 0.01, determined by Student's *t*-test.

Supplementary Figure 6

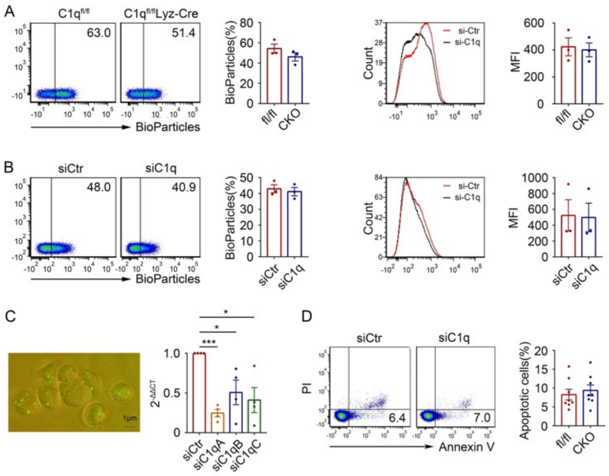

Supplementary Figure 6. C1q deficiency didn't affect phagocytosis and apoptosis of macrophages.

(A) The level of bioparticles phagocytosed by BMDM before and after C1q knockdown (n = 3). (B) The level of bioparticles phagocytosed by BMDM before and after siC1q transfection (n = 3). (C) The transfection efficiency of the 5-Fam (left panel), and C1q expression, quantified by quantitative RT-PCR, was suppressed by siC1q (right panel) (n = 4). (D) The representative flow cytometric dot plots (left panels) and comparisons (right panels) of apoptosis fractions of BMDM before and after C1q knockdown (n = 8). Data were presented as means ± SEM. *P < 0.05, ***P < 0.001, determined by Student's *t*-test.

Supplementary Figure 7

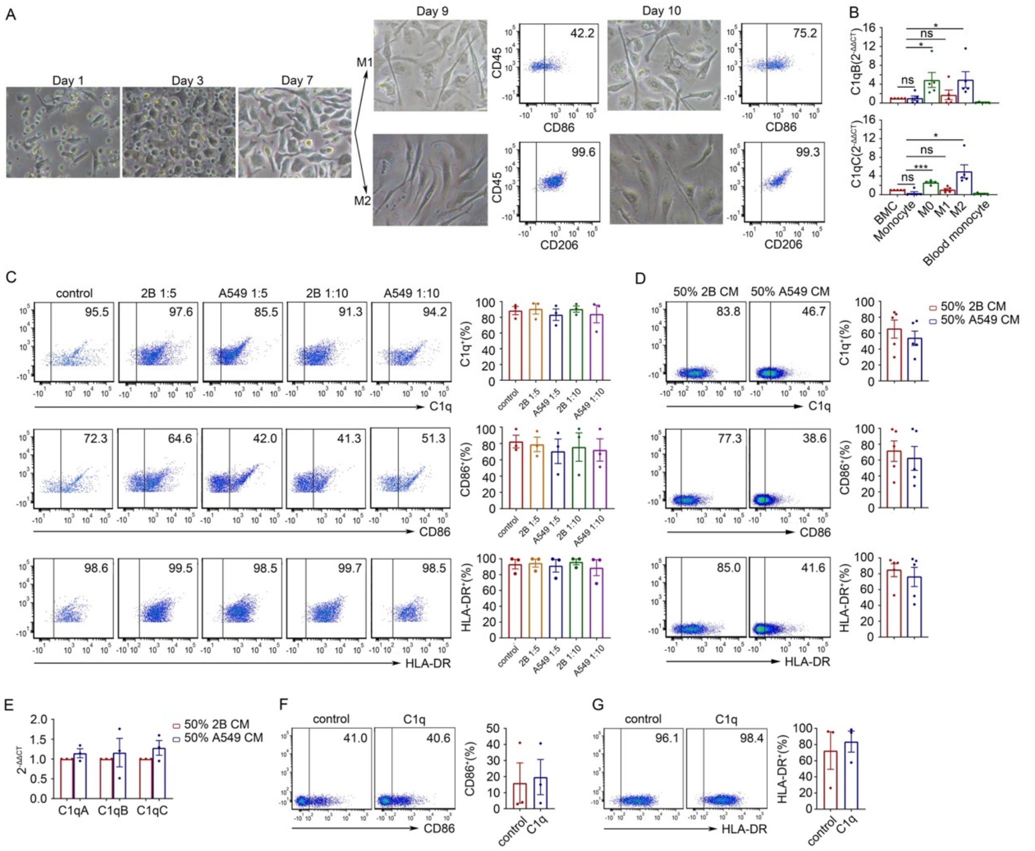

Supplementary Figure 7. C1q affected M2 polarization and tumor environment didn't affect C1q and antigen presentation-related marker gene expression in macrophages from non-MPE patients.

(A) Purified monocytes of bone marrow were incubated for 7 days with macrophage colony-stimulating factor (M-CSF) to differentiate into M0. The M0 were polarized for additional 2 or 3 days into M1 by stimulation with LPS and IFN-γ and into M2 by stimulation with IL-4. (B) mRNA expression of C1q in cells at different stages of bone marrow derived macrophage (BMDM) differentiation was quantified by quantitative RT-PCR (n = 5). (C) $CD14^+$ macrophages isolated from non-MPE and incubated with A549 or 2B cells at different ratios. The level of Mφ C1q, CD86 and HLA-DR were measured after 3 days by flow cytometry. $CD14^+$ macrophages isolated from non-MPE and incubated with conditional medium (CM) of A549 or 2B cells. The level of Mφ C1q, CD86 and HLA-DR were measured after 3 days by flow cytometry (D), and the mRNA level of C1q were measured after 1 day by quantitative RT-PCR (E). $CD14^+$ Mφs isolated from non-MPE and incubated with purified C1q, the level of CD86 (F) and HLA-DR (G) were measured after 3 days by flow cytometry. Data were presented as means ± SEM. *$P < 0.05$ ***$P < 0.001$, determined by Student's *t*-test (D-G) or one-way ANOVA followed by Bonferroni test (B and C)**.**

Supplementary Figure 8

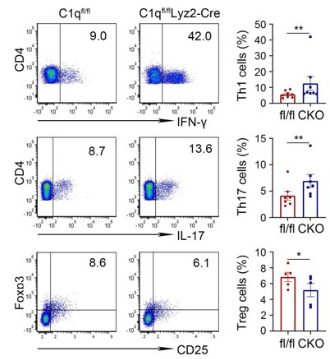

Supplementary Figure 8. C1q deficiency increased Th1 and Th17 cells and inhibited Tregs in MPE.

The representative flow cytometric dot plots (left panel) and statistical comparisons (right panel) of IFN-γ^+, IL-17^+, $CD25^+Foxp3^+$ $CD4^+$ T cells in MPE between $C1q^{fl/fl}$ and $C1q^{fl/fl}$Lyz2-Cre mice receiving intrapleural injection of LLC cells (each n = 5). Data were presented as means ± SEM. *P < 0.05, **P < 0.01, determined by Student's *t*-test.

Supplementary Figure 9

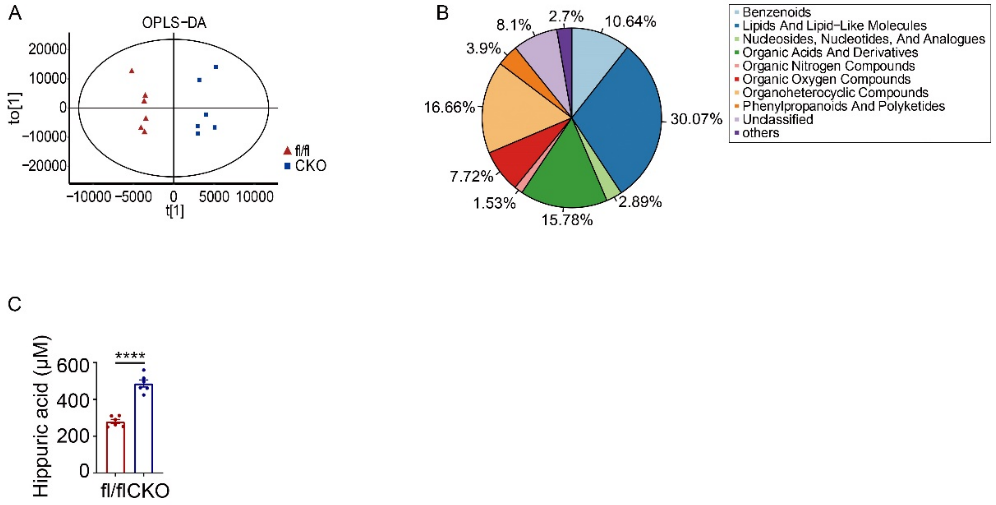

Supplementary Figure 9. Metabolomics analysis of MPE in C1q$^{fl/fl}$ and C1q$^{fl/fl}$Lyz2-Cre mice. (A) Orthogonal PLS-DA (OPLS-DA) of metabolomics profile MPE in C1q$^{fl/fl}$ and C1q$^{fl/fl}$Lyz2-Cre (CKO) mice. (B) The types of metabolites detected by metabolomics. (C) Statistical comparisons of the hippuric acid level in MPE between C1qa$^{fl/fl}$ and CKO mice (n = 6). Data were presented as means ± SEM. ****P < 0.0001, determined by Student's *t*-test.

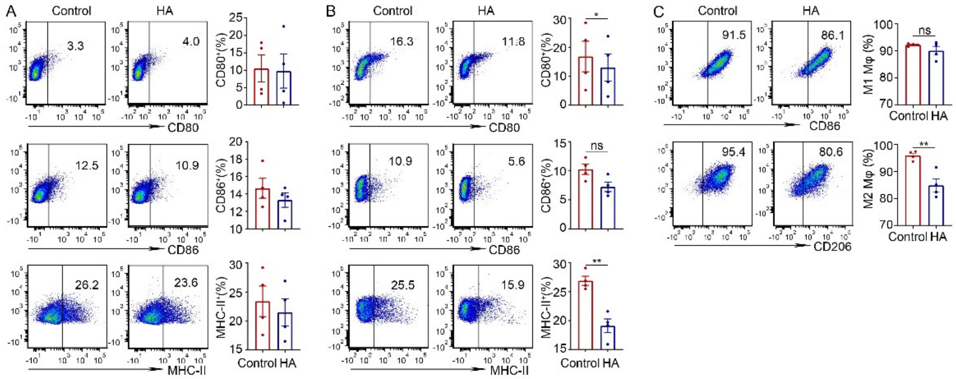

Supplementary Figure 10. Hippuric acid affects the antigen-presenting phenotype of immature macrophages and M2 polarization.

Macrophages isolated from normal pleural cavity (A) and BMDM (B) and treated with hippuric acid or control. The level of CD80, CD86 and MHC II were measured after 3 days by flow cytometry (each $n = 4$). (C) The representative flow cytometric dot plots (left panel) and statistical comparisons (right panel) of M1, M2 macrophages (each $n = 4$). Data were presented as means ± SEM. *$p < 0.05$, **$p < 0.01$, determined by Student's *t*-test.
